# Predicting DNA methylation level across human tissues

**DOI:** 10.1093/nar/gkt1380

**Published:** 2014-01-20

**Authors:** Baoshan Ma, Elissa H. Wilker, Saffron A. G. Willis-Owen, Hyang-Min Byun, Kenny C. C. Wong, Valeria Motta, Andrea A. Baccarelli, Joel Schwartz, William O. C. M. Cookson, Kamal Khabbaz, Murray A. Mittleman, Miriam F. Moffatt, Liming Liang

**Affiliations:** ^1^Department of Epidemiology, Harvard School of Public Health, Boston, MA 02115, USA, ^2^College of Information Science and Technology, Dalian Maritime University, Dalian, Liaoning Province 116026, China, ^3^Cardiovascular Epidemiology Research Unit, Beth Israel Deaconess Medical Center, Boston, MA 02215, USA, ^4^Department of Environmental Health, Harvard School of Public Health, Boston, MA 02115, USA, ^5^National Heart and Lung Institute, Imperial College, London SW3 6LY, UK, ^6^Department of Clinical Sciences and Community, University of Milan, Milan 20122, Italy, ^7^Division of Cardiac Surgery, Department of Surgery, Beth Israel Deaconess Medical Center, Harvard Medical School, Boston, MA 02215, USA and ^8^Department of Biostatistics, Harvard School of Public Health, Boston, MA 02115, USA

## Abstract

Differences in methylation across tissues are critical to cell differentiation and are key to understanding the role of epigenetics in complex diseases. In this investigation, we found that locus-specific methylation differences between tissues are highly consistent across individuals. We developed a novel statistical model to predict locus-specific methylation in target tissue based on methylation in surrogate tissue. The method was evaluated in publicly available data and in two studies using the latest IlluminaBeadChips: a childhood asthma study with methylation measured in both peripheral blood leukocytes (PBL) and lymphoblastoid cell lines; and a study of postoperative atrial fibrillation with methylation in PBL, atrium and artery. We found that our method can greatly improve accuracy of cross-tissue prediction at CpG sites that are variable in the target tissue [*R*^2^ increases from 0.38 (original *R*^2^ between tissues) to 0.89 for PBL-to-artery prediction; from 0.39 to 0.95 for PBL-to-atrium; and from 0.81 to 0.98 for lymphoblastoid cell line-to-PBL based on cross-validation, and confirmed using cross-study prediction]. An extended model with multiple CpGs further improved performance. Our results suggest that large-scale epidemiology studies using easy-to-access surrogate tissues (e.g. blood) could be recalibrated to improve understanding of epigenetics in hard-to-access tissues (e.g. atrium) and might enable non-invasive disease screening using epigenetic profiles.

## INTRODUCTION

Tissue-specific gene expression patterns that determine cell types and functions are regulated in part by tissue-specific methylation at CpG sequences (1). It has been shown that epigenetic variation in the methylation of DNA is related to transcription regulation, cell differentiation, diseases and cancers (2). Recent advances in genome-wide technologies make it possible to study the impact of epigenetics on health outcomes in areas such as cardiovascular epigenetics (3), environmental epigenomics (4), and the role of early life social environment and associations with long-term disorders (5). To understand the variation of methylation in the human genome and its relation to common disease, large-scale population-based studies are needed. However, the target tissues directly relevant to the outcome of interest are often impossible or extremely difficult to collect in a substantial number of samples, which often makes large human studies based on target tissues infeasible. Also, use of DNA methylation data from biospecimens that can be easily and non-invasively collected from human individuals, such as blood, would be critical to develop novel epigenetic biomarkers for clinical diagnosis and prevention. For example, Barault *et al.* (6) showed that leukocyte DNA methylation levels of selected imprinted genes may serve as surrogate markers of DNA methylation in mammary tissue in the study of breast cancer, and Ursini *et al.* (7) showed that methylation in prefrontal cortex target tissue can also be well correlated with methylation in blood lymphocytes.

Previous studies have shown that DNA methylation patterns are largely conserved across tissues, although intra-individual variation exceeds inter-individual variation (2,8,9). For example, Byun *et al.* (2009) reported that the intra-individual correlations for 11 tissues were 0.852 (range: 0.738–0.941) using the IlluminaGoldenGate Bead Array, which integrates 1505 CpG sites in 807 genes. This suggests that it is possible to develop a model to study DNA methylation in target tissues for population-based epidemiological studies using easily accessible tissues. To determine whether methylation markers from surrogate tissues can be used as a proxy for methylation in target tissues to study an outcome of interest, it is necessary to determine whether methylation in surrogate tissues can adequately predict target tissue methylation.

In this study, we systematically addressed this question using the latest high-throughput technologies (Illumina HumanMethylation27 and HumanMethylation450 arrays) to collect data from multiple tissues in the same individuals participating in two independent studies as well as data from public databases. Specifically, first we investigated related tissues including Epstein-Barr virus (EBV)-transformed lymphoblastoid cell lines (LCL) and blood. LCLs have been used to increase the amount of DNA that can be obtained from peripheral blood leukocytes (PBLs) for genetic studies, such as the HapMap (10,11) and the 1000 Genomes Project (12). They have been used to study genetic and epigenetic determinants of gene expression (13–18) and are found to recapitulate the naturally occurring gene expression and methylation variation in primary B- and T cells (8). LCLs are particularly attractive in epigenetic studies because they could potentially allow for DNA methylation analyses even when the amount of blood that can be collected is limited. In the next stage, we examined methylation across differentiated tissues including PBLs, right atrial appendage and left internal mammary artery (subsequently abbreviated as ‘atrium’ and ‘artery’, respectively). We then examined the prediction accuracy using cross-validation and systematically evaluated the performance by predicting methylation status in independent data obtained from a public database repository.

## MATERIALS AND METHODS

### Subject and tissue collection

#### PBL and LCL samples

DNA methylation data were collected from 195 siblings and their parents in 95 nuclear pedigrees identified through a proband with asthma. These data were derived from a previous family study of childhood asthma (19) and gene expression quantitative trait loci mapping exercise for global expression in LCL from a subset of individuals (13). PBL samples were collected from 39 children (18 male) derived from 20 nuclear families collected through a proband with asthma. Among these 39 samples, 22 were asthmatic (see Moffatt *et al.*, 2007, for criteria). DNA from PBLs and paired EBV-transfected LCLs were available from each individual. The transformation of peripheral blood lymphocytes in all 39 samples was carried out by the European Collection of Cell Cultures (ECACC, http://www.hpacultures.org.uk/collections/ecacc.jsp). Previously transformed cryopreserved EBV cell lines were grown as 500-ml roller cultures. Once log phase was reached, cells were pelleted, medium was discarded and a mixture of RLT buffer (RNeasy Lysis Buffer, Qiagen, Valencia, CA, USA) and β-mercaptoethanol was added. Pellets were vortexed to ensure thorough re-suspension, after which they were frozen at −70°C and stored at −80°C (13). DNA was extracted from PBL and LCL using the Promega Wizard Kit.

#### PBL, atrium and artery samples

Patients undergoing coronary artery bypass graft surgery at Beth Israel Deaconess Medical Center were recruited to participate in a study of DNA methylation and atrial fibrillation. PBL, atrium and artery tissue were collected from 18 participants using PaxGene tubes for blood and PaxGene (Qiagen, Valencia, CA, USA) tissue containers. After surgery, six participants developed atrial fibrillation. Blood DNA was extracted using the PAXgene Blood DNA Kit (Qiagen, Valencia, CA, USA); atrial and artery tissue DNA was extracted using the PAXgene Tissue DNA Kit (Qiagen, Valencia, CA, USA) according to the manufacturer’s protocol. Among the four samples with duplicates, correlation *R*^2^ between duplicates was >99%. For 18 participants, one or more tissue types were available. There were 14 individuals with methylation measures in all three tissues that met quality control standards and were included in downstream analysis.

### DNA methylation profiling

#### Illumina Infinium HumanMethylation27 array

DNA samples were quantified using a NanoDrop spectrophotometer (Thermo Scientific, Wilmington, DE, USA) and bisulfite converted using the Zymo EZ DNA Methylation Kit (Zymo Research, Orange, CA, USA) with an input of 1000 ng. The assay was carried out as per the IlluminaInfinium Methylation instructions. Each conversion assay included a commercially available positive control (Universal Methylation DNA Standard, Zymo Research) and in-house–generated negative control (whole-genome amplified genomic DNA). Bisulfite-converted samples were eluted in a volume of 8 μl and re-quantified on the NanoDrop spectrophotometer using the RNA settings (because recovered DNA is single stranded and exhibits similar absorption properties to RNA at 260 nm). Dilution plates were constructed from these bisulfite-converted samples at a concentration of 60 ng/μl in a total volume of 6 μl. These plates (from which 4 μl was ultimately taken) formed the input for the IlluminaInfinium Methylation assay using the HumanMethylation27 BeadChips (IlluminaInc, San Diego, CA, USA). This assay interrogates 27 578 CpG sites for the extent of DNA methylation. The plates were processed as per the manufacturer’s instructions, including the positive and negative controls from each bisulfite conversion assay. Data were visualized using the BeadStudio software, and examined using both sample-dependent and sample-independent quality control criteria. Samples that failed quality control were repeated. Signal intensities of methylated and unmethylated probes were exported from the BeadStudio interface, along with detection of *P*-values representing the likelihood of detection relative to background.

#### Illumina Infinium HumanMethylation450 array

DNA was quantified using a NanoDrop spectrophotometer (NanoDrop Technologies, Wilmington, DE, USA) and PicoGreenQuant-iT TM PicoGreen dsDNA Kit (Invitrogen, Carlsbad, CA, USA). DNA was bisulfite-converted using the Zymo EZ DNA Methylation Kit (Zymo Research, Orange, CA, USA) with an input of 1000 ng using the EZ DNA Methylation Kit (Zymo Research, Orange, CA, USA) according to the manufacturer’s protocol. Final elution was performed with 30 μl M-elution buffer. Bisulfite-treated DNA was aliquoted and stored at −80°C until ready for use. HumanMethylation450 BeadChips (IlluminaInc, San Diego, CA, USA) were used to interrogate ∼450 000 DNA methylation sites covering 14 000 genes including CpG islands and shores, non-coding regions, microRNA promoter, and disease-associated regions plates were processed as per the manufacturer’s instructions, including the positive and negative controls from each bisulfite conversion assay. Data were visualized using the BeadStudio software and were examined using both sample-dependent and sample-independent quality control criteria. Samples that failed quality control were repeated. Signal intensities were exported from the BeadStudio interface both before and after background correction, along with detection *P*-values representing the likelihood of detection relative to background.

### Methylation normalization

#### Methylation β values normalization

For PBL and LCL samples using the HumanMethylation27 array, we normalized the probe intensity by applying quantile normalization to all methylated and unmethylated probes together across all samples, similar to the approach used in the lumi package (*R*, Bioconductor) (20). The methylation β values were recalculated as the ratio of methylated probe signal/(total signal + 100). Individual data points with detection *P* > 0.05 were treated as missing data.

For the PBL, atrium and artery samples using HumanMethylation450 array, we used the pipeline developed by Touleimat and Tost (21). Individual data points with detection *P* > 0.01 or number of beads <3 were treated as missing data. Samples with >20% missing probes were treated as missing data. Probe overlaps with any common single-nucleotide polymorphisms (MAF > 0.05) in the HapMap CEU population and single-nucleotide polymorphisms within 10 bp of query sites were removed. The lumi package (20) was used for background and color bias correction. Quantile normalization across samples was then applied to probes within each functional category (CpG island, shelf, shore, etc.) separately to correct the shift of methylation β value between Infinium I and Infinium II probes by aligning the distribution of Infinium II probes to the reference distribution built on the Infinium probes (21).

### Statistical models for prediction

#### Methylation pattern across tissues

We first examined correlations between PBL and LCL from the asthma study and between PBL, atrium and artery in the atrial fibrillation study. We then removed CpGs with extreme high or low methylation in all samples to assess the correlation between tissues at intermediate level of methylation, as many CpG sites are either completely methylated or unmethylated across individuals and tissues. The first correlations evaluated could be potentially inflated by these CpG sites at both extremes of methylation distribution. This would artificially increase the between-tissue correlation coefficient and mask the relationship at CpGs that shows more tissue specificity.

### Cross-tissue prediction of methylation level

#### Linear prediction model

The linear model was developed using a training data set to predict methylation in a testing data set. Suppose that methylation values in the training data set are organized into two n × m matrices, X and Y, where X is the surrogate tissue and Y is the target tissue. There are n samples and m CpG probes. Each sample is a row and each probe is a column in the matrix. Let *x_ij_* and *y_ij_*, *i* = 1,2, … ,n and *j* = 1,2, … ,m be the element of matrices X and Y, respectively. The linear regression model for prediction of the *j-*th probe is 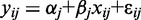, *i* = 1,2, … ,n. Let 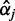 and 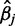 be the estimates of this model. For a particular sample in the testing data set, the predicted methylation value at the target tissue is 
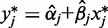
, *j* = 1,2, … ,m, where 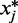 is the methylation value at the surrogate tissue from the sample being predicted.

#### Support vector machine prediction model

Support vector machine (SVM) is one of the most popular supervised learning methods used to analyze data and recognize patterns (22). SVM represents a powerful technique for general (nonlinear) classification, regression and outlier detection and has been widely used in many bioinformatics applications. The SVM function in *R* package e1071 was used to build the statistical prediction model (23). Default parameters for eps-regression were used with radial basis kernel and ε = 0.1. The prediction using the SVM model is constructed in a similar manner as the linear regression method. For a given CpG site *j*, we used *x_ij_* and *y_ij_*, *i* = 1,2, … ,n as the training data set to build an SVM model, denoted as f(x). For a new sample, the predicted value 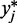 is obtained by applying the SVM model to 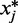, i.e. 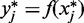, where 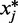 is the methylation value at the surrogate tissue from the sample being predicted. To allow other users to apply our method to their data set, we have prepared an *R* package to do cross-tissue methylation prediction. This *R* package is available to download from our Web site (http://www.hsph.harvard.edu/liming-liang/cross-tissue-methylation/).

#### Assessment of prediction accuracy using cross-validation

Cross-validation was used to estimate prediction accuracy without overfitting. It consists of the following steps:

(i) Leave-one-family out or leave-one-sample out One sample was removed, and the remaining samples were used as training data set. The left-out sample was used as testing data set. Because the PBL–LCL asthma data consist of family members, we removed the entire family in each iteration so that the training data set and the testing data set are completely independent.
(ii) Statistical prediction model (linear regression model or SVM) was estimated using the training data set.
(iii) Applying the prediction model (linear regression model or SVM) to the left-out families or samples, we obtained the predicted value for the samples in testing data set.
(iv) Repeat (i)–(iii) for all families or samples.
(v) After we obtained the predicted value for all n samples and all m CpG sites, 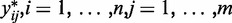. The prediction accuracy was measured by correlation coefficient *R*^2^ and mean absolute error MAE for specific sample (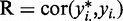, 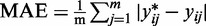) or specific CpG site (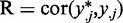, 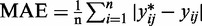), where 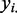 is the *i*th row and 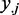 is the *j*th column of the experimentally obtained methylation matrix Y. Similar definition for 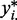 is the *i*-th row and 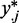 is the *j*-th column of the predicted methylation matrix.

### Single probe versus multiple probes

Methylation at a particular CpG site may be correlated with other CpG sites either at nearby regions or elsewhere on the genome. Including these correlated CpG sites in the prediction model might further improve performance. We examined the utility of including multiple CpG sites in the prediction of atrium methylation based on PBL for 1000 target CpG sites with substantial variation but relatively poor prediction performance (randomly selected from probes with standard deviation in atrium between 0.1 and 0.2, and *R*^2^ between PBL and atrium <0.3).

### Prediction generalizability across studies

We further evaluated the prediction performance by applying our prediction model to an independent data set described in Caliskan *et al.* (2011) (GEO accession ID: GSE26211). This data set contains six individuals. Each individual has two T cell samples and 12 LCL samples (24 LCL-T cell pairs for each individual). For each LCL-T cell pair, we applied the SVM and linear model built using our 39 LCL-PBL samples to the LCL sample and compared the predicted value with the T cell methylation value.

### Prediction performance in other tissue settings

To evaluate our model performance in other tissues, we have obtained data from Byun *et al.* (2009) (2), where there are six cases and each has 11 tissues: brain, bladder, colon, esophagus, heart, kidney, liver, lung, pancreas, spleen and stomach. We examined all tissue pairs (55 pairs). For each pair, we applied our SVM model to predict methylation in one tissue using the other tissue in the pair.

### Cross-tissue prediction and utility of surrogate tissue

To examine the utility of the predicted methylation value, we carried out association analysis between postoperative atrial fibrillation (PostOpAF, the outcome of primary interest) with the following linear regression model:

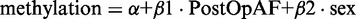

where outcome is the methylation of individual probe and PostOpAF is the postoperative atrial fibrillation. We then performed a clustering analyses using PBL, atrium and predicted atrium methylation to evaluate hierarchical clustering by AF status based on peripheral blood leukocytes and tissue methylation as well as predicted methylation levels.

### Sample size effect on prediction accuracy

We hypothesized that a training data set with a larger sample size would increase the precision of model parameters and reduce the effect of outliers. We evaluated the sample size effect by randomly choosing a subset of our 39 PBL-LCL samples as training data set and predicted the methylation in the GSE26211 data set. We varied the size of the training data set from 3, 4, 5, 6, 7, 8, 10, 20, 30 to 39. For each size of the training data set (except *n* = 39), we randomly selected a different training data set 10 times and reported the average performance across the 10 replicates.

## RESULTS

### Methylation pattern across tissues

Consistent with previous studies (2,8), we also observed that DNA methylation values were largely conserved across tissues. The correlation *R*^2^ between PBL and LCL for the 39 samples ranged from 0.81 to 0.95 with mean 0.92 (Supplementary Figure S1 and W-S1). In the 14 atrial fibrillation study (AF) samples, the correlation between tissues was substantially high (for PBL-artery, *R*^2^ ranged from 0.76 to 0.89 with mean 0.81, for PBL-atrium, *R*^2^ ranged from 0.81 to 0.87 with mean 0.83, for artery-atrium, *R*^2^ ranged from 0.91 to 0.97 with mean 0.94, Supplementary Figure S2 and W-S2). After removing CpG sites with minimum methylation β value > 0.9 or maximum β value < 0.1 among all subjects and tissues, correlations were reduced. They were further reduced after removing CpG sites with minimum methylation β values >0.8 or maximum β values <0.2 among all subjects and tissues (Table1).

**Table 1.** Correlation R^2^ between raw data across tissues in asthma study and AF study

Tissue pair	All probes		Remove probes with minimum methylation β value > 0.9 or maximum β value < 0.1			Remove probes with minimum methylation β value > 0.8 or maximum β value < 0.2		
	Mean correlation	range	Mean correlation	Range	Probes removed	Mean correlation	Range	Probes removed
Illumina27k data from asthma study								
PBL-LCL	0.92	0.81, 0.95	0.88	0.71, 0.92	10 543	0.81	0.53, 0.87	15 463
Illumina450k data from AF study								
PBL-artery	0.81	0.76, 0.89	0.59	0.48, 0.75	174 124	0.38	0.25, 0.61	248 496
PBL-atrium	0.83	0.81, 0.87	0.61	0.57, 0.70	179 645	0.39	0.33, 0.51	257 563
Atrium-artery	0.94	0.91, 0.97	0.84	0.76, 0.91	194 004	0.71	0.59, 0.84	271 094

For ‘All probes’ column, we used the raw methylation to calculate *R*^2^. For ‘Remove probes with min methylation β value > 0.9 or max β value < 0.1’ column, we removed the extreme probes those fall within this range and used the remaining data to calculate R^2^, and it is similar to ‘Remove probes with minimum methylation β value > 0.8 or maximum β value < 0.2’ column.

Despite the high level of overall correlation *R*^2^, there were many CpG sites that exhibited differences in methylation across tissues (14% CpGs have methylation difference >0.1 in PBL-LCL data set, 26% in PBL-artery, 24% in PBL-atrium and 14% in artery–atrium data sets).These CpG sites determined tissue-specific methylation patterns. We first examined how cross-tissue differences in methylation at each locus were distributed across individuals and found that they were largely consistent. For example, a CpG site that had higher methylation level in target tissue than surrogate tissue in one individual generally had higher methylation level in target tissue than surrogate tissue in other individuals. In addition, the magnitude of difference was similar across individuals (Supplementary Figure S3, S4, W-S3 and W-S4).

### Cross tissue prediction of methylation level

We explored the utility of methylation prediction by using two statistical models based on linear regression model (LM) and SVM and two independent data sets with five tissues. Leave-one-out cross-validation procedure is used to estimate the prediction accuracy and avoid overfitting.

#### PBL and LCL

**Figure 1.**
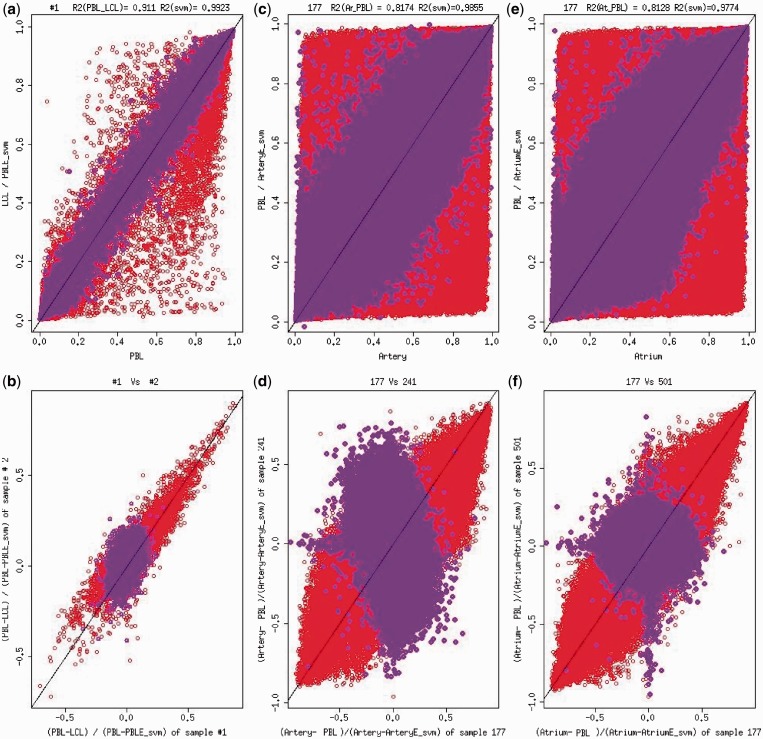
Methylation pattern across tissues and between-tissue difference across individuals. (**a**) Scatter plots for sample #1. Red circles for PBL versus LCL (x = PBL, y = LCL) and purple circles for PBL versus SVM-predicted PBL (x = PBL, y = predicted PBL based on LCL). R^2^ (PBL_LCL) = R^2^ between methylation in PBL and methylation in LCL. R^2^(SVM) = R^2^ between methylation in PBL and SVM predicted methylation in PBL based on LCL data. (**b**) Scatter plot for sample #1 versus sample #2. Red circles for PBL–LCL in sample #1 versus PBL–LCL in sample #2 (x = PBL–LCL in sample #1, y = PBL–LCL in sample #2). Purple circles for PBL–SVM-predicted PBL of sample #1 versus PBL–SVM-predicted PBL in sample #2 (x = PBL–SVM-predicted PBL of sample #1, y = PBL–SVM-predicted PBL of sample #2). (**c**) Scatter plots for sample 177. Red circles for Artery versus PBL (x = artery, y = PBL) and purple circles for artery versus SVM-predicted artery (x = artery, y = predicted artery based on PBL). R^2^(Ar_PBL) = R^2^ between methylation in artery and methylation in PBL. R^2^(SVM) = R^2^ between methylation in artery and SVM-predicted methylation in artery based on PBL data. (**d**) Scatter plot for sample 177 versus sample 241. Red circles for artery–PBL in sample 177 versus artery–PBL in sample 241 (x = artery–PBL in sample 177, y = artery–PBL in sample 241). Purple circles for artery–SVM-predicted artery of sample 177 versus artery–SVM-predicted artery in sample 241 (x = Artery–SVM-predicted artery of sample 177, y = artery–SVM-predicted artery of sample 241). (**e**) Scatter plots for sample 177. Red circles for atrium versus PBL (x = atrium, y = PBL) and purple circles for atrium versus SVM-predicted atrium (x = atrium, y = predicted atrium based on PBL). R^2^(At_PBL) = R^2^ between methylation in atrium and methylation in PBL. R^2^(SVM) = R^2^ between methylation in atrium and SVM-predicted methylation in atrium based on PBL data. (**f**) Scatter plot for sample 177 versus sample 501. Red circles for atrium–PBL in sample 177 versus atrium–PBL in sample 501 (x = atrium–PBL in sample 177, y = atrium–PBL in sample 501). Purple circles for atrium–SVM-predicted atrium of sample 177 versus Atrium–SVM-predicted atrium in sample 501 (x = atrium–SVM-predicted atrium of sample 177, y = atrium–SVM-predicted atrium of sample 501). Asterisk: for scatter plots for all other samples; please refer to Supplementary Figures S5 and W-S5 for LCL-PBL, S6 and W-S6 for PBL–artery, S7 and W-S7 for PBL–atrium.

After iterating through all 20 families, we had a vector of 39 predicted methylation levels in PBL and a vector of their observed methylation levels as measured by the Illumina array. Correlation *R*^2^ between these two vectors was used to evaluate the prediction performance for the CpG site (CpG-specific or probe-specific accuracy). We applied this leave-one-out procedure on all probes of the Illumina array and obtained a predicted methylation vector for all CpG sites for each sample. Results showed that the predicted PBL methylation level was much closer to its experimental counterpart (methylation measured directly in PBL). The improvement was illustrated through the scatter plots of LCL versus PBL and predicted PBL versus PBL for sample #1 in Figure 1a (based on SVM prediction) and Supplementary Figure S8a (based on LM prediction). The difference between predicted PBL and PBL is greatly reduced and consistent across samples (Table 2, Figure 1b and Supplementary Figure S8b for sample #1 and #2). Similar improvement was observed for all 39 samples (Supplementary Figure S5 and W-S5) and the overall correlation *R*^2^ between true and predicted PBL methylation from the same individual increased from 0.92 to 0.99 (average across all 39 samples for both linear regression model and SVM model). After eliminating CpG sites that were completely methylated or unmethylated, we still observed substantial increases in the *R*^2^. The mean absolute error is mostly below one standard deviation of methylation in PBL. Smaller error was observed for probes with large variation.

**Table 2.** Mean correlation R^2^ between true and predicted methylation in asthma study and AF study

	All probes	Remove probes with minimum methylation β value > 0.9 or maximum β value < 0.1	Remove probes with minimum methylation β value > 0.8 or maximum β value < 0.2
Illumina27k data from asthma study			
PBL-predicted PBL	0.99	0.99	0.98
Illumina450k data from AF study			
artery-predicted artery	0.97	0.93	0.89
atrium-predicted atrium	0.99	0.97	0.95

For the first column, we calculated the mean *R*^2^ using true methylation and predicted methylation across all samples. For the second column, we removed the extreme probes those fall within the range of minimum methylation β value > 0.9 or maximum β value < 0.1, and then calculated the mean *R*^2^ using the remaining data across all samples. It is similar to the last column.

#### PBL, artery and atrium

**Figure 2.**
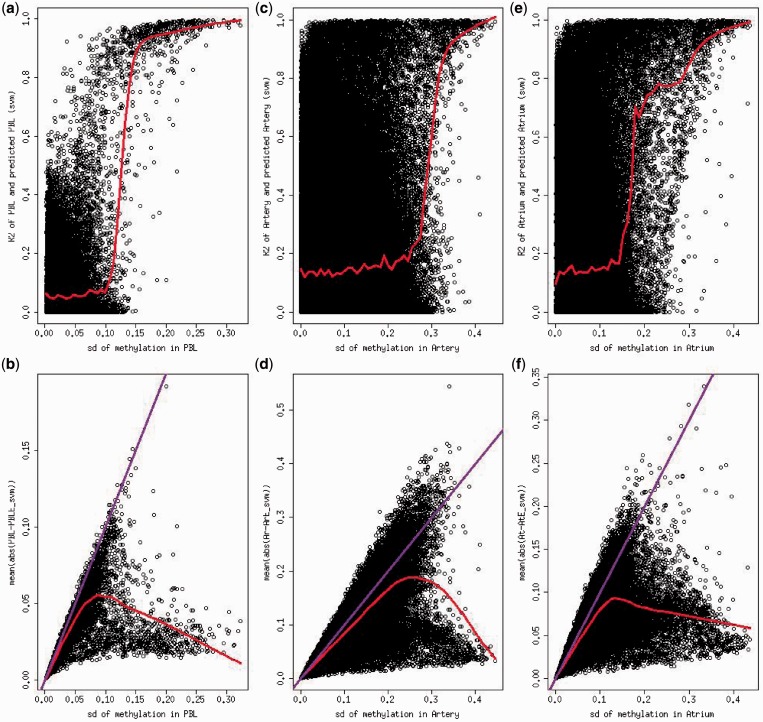
Probe-specific prediction accuracy based on SVM model by methylation variation within target tissues. (**a**) Standard deviation (SD) of methylation in PBL versus *R*^2^ between PBL and predicted PBL based on SVM. For each dot, x = standard deviation (SD) of methylation in PBL and y = the R^2^ of PBL and predicted PBL using SVM model for the same probe. (**b**) Standard deviation (SD) of methylation in PBL versus mean absolute value of difference between PBL and predicted PBL based on SVM. For each dot, x = standard deviation (SD) of methylation in PBL and y = the mean absolute value of difference between PBL and predicted PBL using SVM model for the same probe. (**c**) Standard deviation (SD) of methylation in artery versus *R*^2^ between artery and predicted artery based on SVM. For each dot, x = standard deviation (SD) of methylation in artery and y = the R^2^ of artery and predicted artery using SVM model for the same probe. (**d**) Standard deviation (SD) of methylation in artery versus mean absolute value of difference between artery and predicted artery based on SVM. For each dot, x = standard deviation (SD) of methylation in artery and y = the mean absolute value of difference between artery and predicted artery using SVM model for the same probe. (**e**) Standard deviation (SD) of methylation in atrium versus *R*^2^ between atrium and predicted atrium based on SVM. For each dot, x = standard deviation (SD) of methylation in atrium and y = the R^2^ of atrium and predicted atrium using SVM model for the same probe.(**f**) Standard deviation (SD) of methylation in atrium versus mean absolute value of difference between atrium and predicted atrium based on SVM. For each dot, x = standard deviation (SD) of methylation in atrium and y = the mean absolute value of difference between atrium and predicted atrium using SVM model for the same probe. Asterisk: each dot represents one probe on the Illumina array. The curve represents the LOESS smoothing average curve. The straight line in (b), (d) and (f) is the x = y line.

The relatively close relationship between PBL and LCL methylation might contribute to the good prediction accuracy. We next extended the same prediction procedures (linear regression model and SVM model) to the second data set where three tissues (atrium, internal mammary artery and PBL) using IlluminaInfinium HumanMethylation450 array were collected from 14 individuals. We treated PBL as the surrogate tissue and artery and atrium as the target tissues. The correlations between artery-raw PBL (*R*^2 ^= 0.81) and atrium-raw PBL (*R*^2 ^= 0.83) are less than PBL-LCL but are similar to correlations reported in other studies (2). Again, we observed that the predicted artery or atrium methylation level is much closer to its experimental counterpart (Figure 1c–f and Supplementary Figures S6, S7, S8c–S8f, W-S6, W-S7) and the overall correlation *R*^2^ increased from 0.81 (raw PBL-artery) to 0.97 (calibrated PBL-artery) or from 0.83 (raw PBL-atrium) to 0.99 (calibrated PBL-atrium). After removing CpG sites with minimum methylation β value > 0.9 or maximum β value < 0.1 among all subjects and tissues, our prediction model substantially increased the overall *R*^2^ (Table 2). At individual CpG sites, we observed that when there is substantial variation [SD > 0.35 (LM)], SD > 0.33 (SVM) for artery or SD > 0.27 (LM), SD > 0.30 (SVM) for atrium in the target tissue (artery or atrium), the prediction accuracy is close to 1, and the mean absolute error is mostly below 1 SD of methylation in the target tissues (Figure 2c–f and Supplementary Figure S9c–S9f).

**Figure 3.**
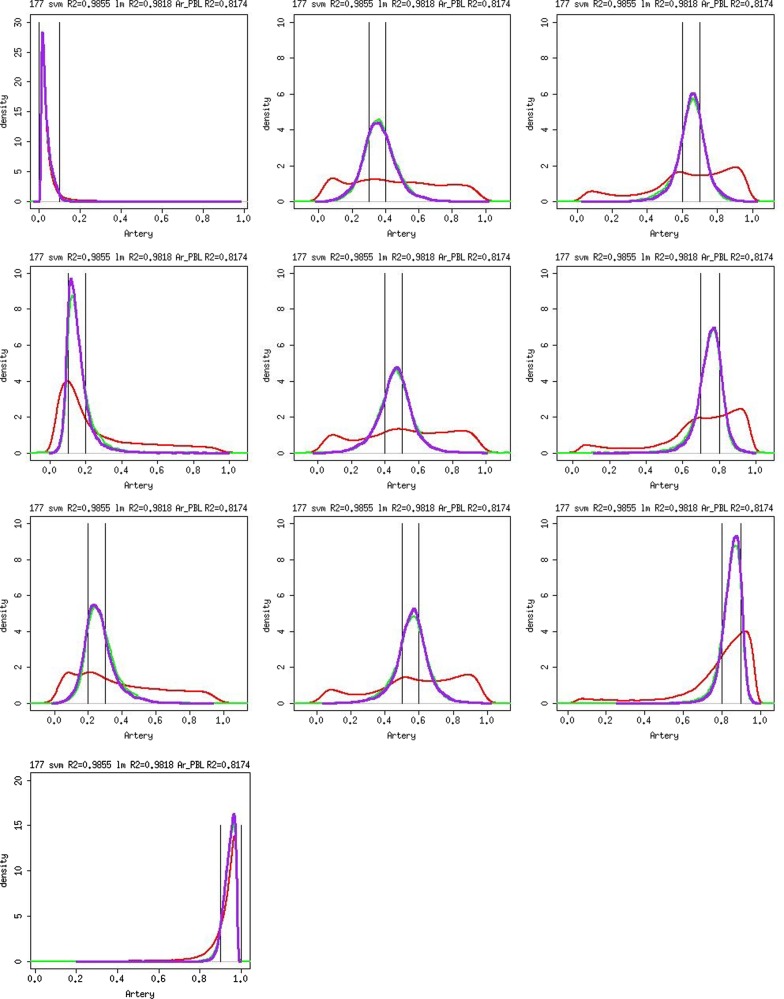
Density of predicted methylation level by true methylation in artery for sample 177. Asterisk: red line represents the density of methylation in PBL. Green line represents the density of the predicted artery methylation by using linear regression model. Purple line represents predicted methylation using SVM model. The two vertical lines represent the range of true methylation level in artery.

For some individuals, the scatter plots in Figure 1c and e and Supplementary Figure S8c, S8e become less informative due to the large spread of data points. Therefore, we examined the density of predicted values by different intervals of experimentally obtained methylation values. For example, we categorized the probes according to artery methylation values from one participant into 10 equally spaced bins and plotted the density of predicted values for probes within each bin (Figure 3). Our analysis shows that the predicted values by either model were more likely to fall within the window of the experimentally obtained methylation level than uncalibrated methylation level measured in PBL. The density plots from another individual show similar pattern (Supplementary Figure S10). These results suggested that the predicted artery, atrium or calibrated PBL methylation is a better surrogate model than the raw PBL methylation level to study methylation variation in artery/atrium. Although the original correlation is >0.8 based on all probes, the calibrated values still gave higher correlation *R*^2^ (0.97 for PBL-artery prediction and 0.99 for PBL-atrium prediction, both linear regression model and SVM model).

#### Linear regression versus SVM

We have used both linear regression model and SVM model for our prediction engines. These two models gave similar overall performance (Figure 1 and Supplementary Figure S8). We expected that the linear regression model would be more vulnerable to outliers for small sample size. After examining the range of predicted values, we found that the linear regression model can sometimes produce methylation values out of the 0–1 range due to the effect of outliers and extrapolation, whereas SVM regression always gives prediction within the range spanned by the training data set (Supplementary Figures S11, W-S8, S12, W-S9, S14 and S15). For the larger PBL–LCL sample, the influence of outliers is reduced (Supplementary Figures S13, W-S10, S16).

### Prediction generalizability across studies

In the independent data set of six individuals and 24 LCL T cell pairs, the correlation *R*^2^ between predicted value and T cell methylation was 0.95 for both SVM and LM models (average across 144 LCL-T cell pairs) compared with 0.92 for LCL-T cell correlation (Supplementary Figure S17). After removing CpG sites with minimum methylation β values > 0.9 or maximum β values < 0.1 among all subjects in both LCL and T cells, our prediction model increased the overall *R*^2^ from 0.88 to 0.92. When the cut-points for minimum and maximum β values were changed to 0.8 and 0.2, respectively, the overall correlation *R*^2^ increased from 0.80 to 0.87. The magnitude of improvement was smaller than the cross-validation estimate in our 39 samples (Supplementary Figure S17). This is likely because the target tissue in the training data set is PBLs, whereas the target tissue in testing data set is T cells. The significant improvement, especially for LCL-T cell pairs with lower correlation (Supplementary Figure S17), suggests that the prediction model built in a training data set is applicable to future studies and improves the utility of surrogate tissues.

### Prediction performance in other tissue settings

**Figure 4.**
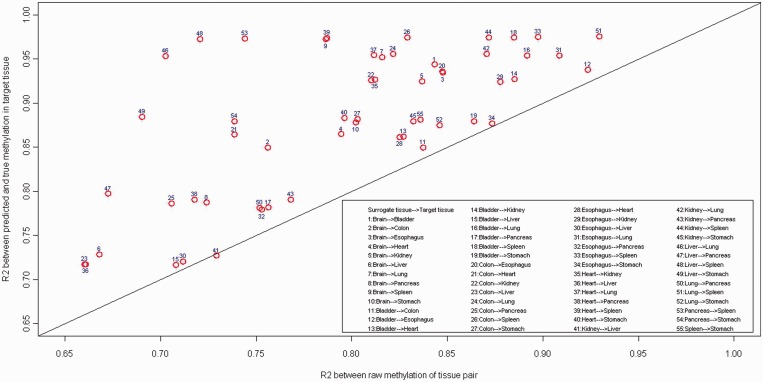
Predicting performance across multiple tissues. Data obtained from Byun *et al.* (2009) Hum Mol Genet (PMID:19776032), where there are six cases and each has 11 tissues: brain, bladder, colon, esophagus, heart, kidney, liver, lung, pancreas, spleen and stomach. We examined all tissue pairs (55 pairs). For each pair, we apply our SVM model to predict methylation in one tissue using the other tissue in the pair. Figure 4 compares the R^2^ based on raw data and predicted data. R^2^ of raw data is the R^2^ between raw methylation of tissue pair by individual and average across all six subjects, R^2^ of predicted data is the R^2^ between predicted and true methylation in the target tissue by individual and average across all six subjects. The straight line is the x = y line. In the legend, the surrogate tissue is on the left and target tissue is on the right.

To evaluate our model performance in other tissues, we obtained data from Byun *et al.* 2009 (2), where there were six cases with 11 tissues: brain, bladder, colon, esophagus, heart, kidney, liver, lung, pancreas, spleen and stomach. We examined all tissue pairs (55 pairs). For each pair, we applied our SVM model to predict methylation in one tissue using the other tissue in the pair. Figure 4 compares the *R*^2^ based on raw data and predicted data. *R*^2^ of raw data is the *R*^2^ between raw methylation of tissue pair by individual and average across all six subjects, *R*^2^ of predicted data is the *R*^2^ between predicted and true methylation in the target tissue by individual and average across all six subjects. Our results demonstrate that cross-tissue methylation prediction is feasible and its performance depends on actual tissue pairs and sample size. In the future, collection of additional paired tissue data could be used to re-train the prediction model, and would be useful for other large-scale study based on blood, which was not included in this study.

### Cross-tissue prediction improves utility of surrogate tissue

**Figure 5.**
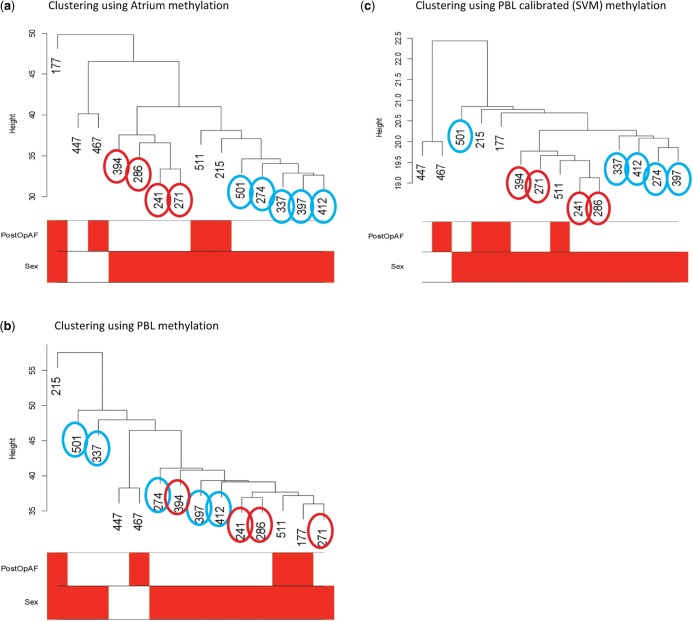
Clustering using atrium, PBL and PBL calibrated (SVM) methylation. (**a**) There are 14 samples: two female (white) and 12 male (red). The PostOpAF contains four cases (red) and 10 controls (white). The controls are grouped into two groups indicated by red circles and turquoise circles. One group contains sample #394, #286, #241, #271 and the other group includes #501, #274, #337, #397 and #412. (**b**) The two groups of controls indicated by red and turquoise circles are mixed together and one control (#271) is first clustered with two cases (#511, #177) and then with other controls (turquoise and red), and there are two controls (#501, #337) distinct from other controls. (**c**) The turquoise and red controls are now clustered back together, respectively, and locate at the bottom of the tree, except control #501 that was also close to case #215 using atrium methylation (a) and case #511 that is now clustered with the red group controls but was clustered with turquoise controls.

Using effect size for AF derived from atrium methylation as the gold standard, we found that predicted atrium methylation (or calibrated PBL methylation) gave less bias in the effect size than uncalibrated PBL methylation in 60% of loci based on SVM (59% based on LM). We anticipate that improvement will increase with larger sample size. We performed a clustering analyses using PBL, atrium and predicted atrium methylation by SVM (see Figure 5). When we cluster by atrium methylation, the controls are clustered in two groups (Figure 5a). The clustering by PBL methylation shows a different pattern and cases and controls cluster together. When we use the predicted atrium methylation, we find that it better represents the patterns showing from the true atrium methylation data, thus improving the utility of the PBL tissue as a surrogate of the atrium tissue. This result suggests the predicted methylation can be particularly useful for analyses that involve multiple CpGs, such as the clustering analysis here and network analyses that show to be useful for gene expression data (24,25).

### Sample size effect on prediction accuracy

**Figure 6.**
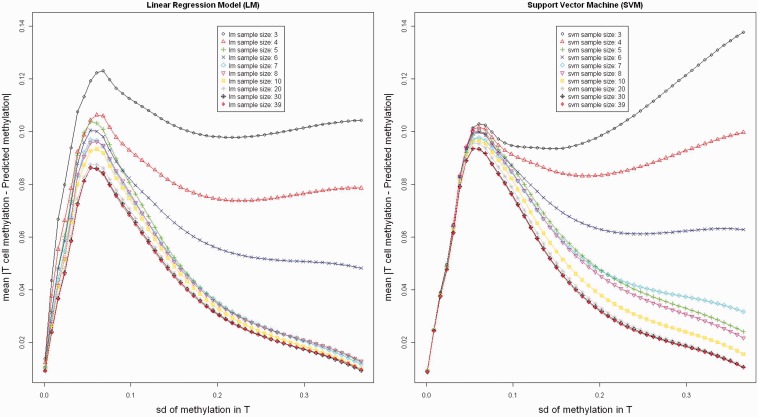
Effect of training sample size on cross-study probe-specific prediction error. Asterisk: for a given sample size, we randomly chose samples from our family data set of 39 individuals to construct the training sample and predict the T cell methylation in the GSE26211 data set. We replicated this 10 times and computed the mean absolute prediction error for each probe. The prediction error is plotted against standard deviation of methylation in the target tissue (T cell methylation). The left panel is prediction error by using linear regression model; the right panel is prediction error by using SVM model.

For each of the six individuals in the GSE26211 data set, there are 24 LCL-T cell pairs. For each LCL-T cell pair, we computed the average absolute difference across all probes as prediction accuracy for the pair. In Supplementary Figure S18, each line represents a particular LCL-T cell pair in one of the 10 replicates. The trend of the lines shows that increasing sample size increases overall prediction accuracy for the sample. SVM has a generally better performance than the linear model and is less subjective to influential outliers (the pairs with poor prediction accuracy were all based on one LCL sample’s prediction of T cells from the same individual, purple lines at left panel). We also examined probe-specific prediction accuracy. Increasing sample size can greatly reduce prediction errors, especially when the methylation has substantial variation (Figure 6).

We observed that prediction error rate was higher with methylation variation at the beginning and then decreased dramatically. We speculate that a relatively constant technical measurement error in methylation across probes could possibly explain the increasing of prediction error when total variance is small, which is dominated by technical measurement errors. But this hypothesis needs to be tested with additional experiments.

When the training data set was too small (*n* = 3 or 4), the training samples were not able to represent the majority of the population. Consequently, prediction error increased along with the methylation variance, particularly for SVM, which ensures that the prediction value falls within the range of the training samples (Figure 6). We recommend that at least 10 samples are used in the training data set, and this could vary by tissues and the technology used to measure methylation.

## DISCUSSION

In this study, we developed and systematically evaluated statistical models to predict methylation level at target tissues using surrogate tissues. Through both cross-validation and the application to an independent data set, we showed that methylation value at the target tissue can be well predicted, especially for CpG sites with substantial variation in the target tissue. It suggests that one can improve the utility of surrogate tissues by learning the relationship between target tissues and surrogate tissues in an independent data set. We expect that the prediction accuracy could vary across tissue type and different populations. Predictions may be further improved by incorporating more information, such as from correlated CpG sites and additional samples as discussed below.

### Prediction accuracy on independent samples

The cross-validation procedure estimates the prediction accuracy for target tissue of an independent sample that was drawn from the same study population and was processed and normalized together with the surrogate tissue. When the training data set and study data set were from different cell populations, we expect the prediction accuracy will be lower than the estimate from cross-validation as was seen in the prediction exercise using the GSE26211 samples (target tissue is PBL in training data versus target tissue is T cell in testing data). To maximize prediction accuracy, it is ideal to collect the training sample from the cell type as close to the target cell type as possible and use the same technology platform for the methylation data.

### Potential limitations of cross-tissue prediction

We expect there are limitations in applying cross-tissue prediction in epidemiology studies. It is possible that correlation between tissues might not necessarily apply to the CpG sites that are informative for a specific phenotype or disease. In situations when an important exposure only affects the methylation in the target tissue but has little impact on the surrogate tissue, the cross-tissue prediction might not work well. Genotypes that destroy, introduce or shift a CpG would lead to correlation between tissues. The accurate prediction of methylation would reflect the prediction of genotype in this case. Environmental exposures applied to both tissues, especially at the developmental stage, would also lead to high correlation between tissues. In general, processes unique to diseased tissues would make the prediction become difficult.

In the atrial fibrillation study, we have four cases and 10 controls. For the four cases, the average *R*^2^ between raw PBL and atrium methylation is 0.83, whereas average *R*^2^ between atrium and predicted atrium methylation by the SVM model is 0.98. For the 10 control samples, the average *R*^2^ between raw PBL and atrium methylation is 0.83, whereas the average *R*^2^ between atrium and predicted atrium methylation by the SVM model is 0.99. In our data set, the prediction performance was similar in cases and controls. If the relationship across tissues differs in cases and controls, it would be required to include both case and control subjects in the training data set. We recommend that it is important for the training data set to cover as many important conditions as possible. In future work, we will extend our model to explicitly take into account such information, e.g. model the relationship separately in cases and controls and incorporate environmental exposure information.

### Implications to epidemiology study and clinical utility

This study confirmed the finding from previous studies that methylation level is largely conserved across tissues and showed that methylation status measured in surrogate tissues can be further recalibrated to better represent the true methylation status in target tissues, which would greatly enhance the potential utility of the surrogate model. The utility of this method will depend on the actual tissue pairs and sample size of the training data set, as demonstrated in Figures 4 and 6. We note that our method can be used to evaluate the usefulness of a proposed surrogate tissue for a specific target tissue. It would be important for a pilot study to evaluate the feasibility for a large-scale study to use the surrogate tissue. If the surrogate tissue would be representative of the target tissue, our method provides a way to greatly improve the utility of the surrogate tissue, as we have shown the predicted value is a much better surrogate than the original raw methylation value. With the proposed methylation recalibration or prediction, large-scale epidemiological studies could become feasible if surrogate tissue, such as blood, is the only available data.

The clinical utility of methylation markers identified in surrogate tissues could be improved by using our method to calibrate the methylation level to better represent the status in target tissues. For example, in the study for postoperative atrial fibrillation, our results suggest that atrium epigenome might be informative to predict postoperative atrial fibrillation (which presents in ∼30% of the patients). The lower accuracy in artery compared with atrium is likely because the artery tissues collected is in fact a mixture of endothelium, blood and smooth muscle cells, thus increasing noise in the target methylation level. If we could identify patients with at-risk epigenomes, we could treat them with prophylactic therapy or intensive mornitoring. Also, if prediction could be done with blood rather than atrium, clinical strategy could be developed in advance of the surgery. We have summarized potential applications of our approach in Table 3 along with strengths and limitations. This list is not meant to be complete but could provide some guideline as how to better use surrogate tissue in large-scale epidemiology studies.

**Table 3.** Potential applications of the cross-tissue recalibration approach

Potential applications	Strengths	Limitations
Evaluate of the utility of surrogate tissues before study is undertaken	Useful for study design to choose appropriate tissue and evaluation of feasibility	CpGs with high correlation may not be disease relevant
Improve utility of surrogate tissues after study is undertaken	Improve statistical power and obtain unbiased effect estimate in data analysis based on collected samples	Efficiency may depend on size of available training data
Pre-identify useful surrogate tissues for a series of target tissues based on a public database with large samples and large number of tissues available	Useful for study design when paired tissue cannot be obtained within the study	Correlation between tissues might be study/population specific. Correlations found in healthy subjects might not apply to diseased individuals.
Identify candidate genes that can be represented in surrogate tissues	Candidate gene of interest may have high correlation between tissues even if other genes are not	Candidate genes of interest may not be well covered by a particular platform
Estimate the sample size required for the training data set	Useful for study design to collect enough samples for the training data set	Need pilot study or public data to estimate overall correlation between tissues

## SUPPLEMENTARY DATA

Supplementary Data are available at NAR Online.

## FUNDING

HSPH-NIEHS center [ES000002], NIH [T32-HL007374], NIH [R01GM104411], NIH [R01ES021733] postdoctoral research fellow scholarship of the China Scholarship Council [2011821109], Fundamental Research Funds for the Central Universities and Young Foundation of Dalian Maritime University [2011QN119]. Funding for open access charge: NIH [R01GM104411] and NIH [R01ES021733].

*Conflict of interest statement*. None declared.

## Supplementary Material

Supplementary Data
