# Supplementary material for: Predicting DNA methylation level across human tissues
Source: Nucleic Acids Res. 2014 Jan 20;42(6):3515–28. doi: 10.1093/nar/gkt1380 (PMC3973306; doi:10.1093/nar/gkt1380)
Supplement: Supplementary Data [file supp_42_6_3515__index.html]

Predicting DNA methylation level across human tissues — Predicting DNA methylation level across human tissues — Supplementary Data 

# Predicting DNA methylation level across human tissues

## Supplementary Data

files

**Files in this Data Supplement:**

- Supplementary Data - pdf file
