# Supplementary material for: Predicting DNA methylation level across human tissues
Source: Nucleic Acids Res. 2014 Jan 20;42(6):3515–28. doi: 10.1093/nar/gkt1380 (PMC3973306; doi:10.1093/nar/gkt1380)
Supplement: Supplementary Data [file supp_gkt1380_nar-02566-n-2013-File002.pdf]

Supplementary Figure Legends:

Supplementary S1. Scatter plot of LCL and PBL of sample 1 to 9

Supplementary S2. Scatter plot of PBL, Artery and Atrium of sample 1 to 9

Supplementary S3. Scatter plot of PBL - LCL of sample 1 to 9

Supplementary S4. Scatter plot of Artery - PBL, Atrium - PBL and Atrium - Artery of sample 1 to 9

Supplementary S5. Scatter plot of LCL, PBL and predicted PBL (LM, SVM) of sample 1 to 9

Supplementary S6. Scatter plot of PBL, Artery and predicted Artery (LM, SVM) of sample 1 to 9

Supplementary S7. Scatter plot of PBL, Atrium and predicted Atrium (LM, SVM) of sample 1 to 9

Supplementary S8. Methylation pattern across tissues and between-tissue difference across individuals

Supplementary S9. Probe specific prediction accuracy based on LM model by methylation variation within target tissues

Supplementary S10. Density of predicted methylation level by true methylation in Artery for sample 271

Supplementary S11. Scatter plot of PBL, Artery and predicted Artery (LM, SVM) of sample 1 to 9

Supplementary S12. Scatter plot of PBL, Atrium and predicted Atrium (LM, SVM) of sample 1 to 9

Supplementary S13. Scatter plot of LCL, PBL and predicted PBL (LM, SVM) of sample 1 to 9

Supplementary S14. Scatter plot of PBL, Artery and predicted Artery (LM, SVM) of sample 215

Supplementary S15. Scatter plot of PBL, Atrium and predicted Atrium (LM, SVM) of sample 215

Supplementary S16. Scatter plot of LCL, PBL and predicted PBL (LM, SVM) of sample 8

Supplementary S17. R2 of LCL-PBL dataset and GSE26211 dataset

Supplementary S18. Sample size effect on individual specific prediction error

Note: please download Supplementary W-S1 to W-S10 from our website:
http://www.hsph.harvard.edu/liming-liang/cross-tissue-methylation/

Supplementary S1. Scatter plot of LCL and PBL of sample 1 to 9

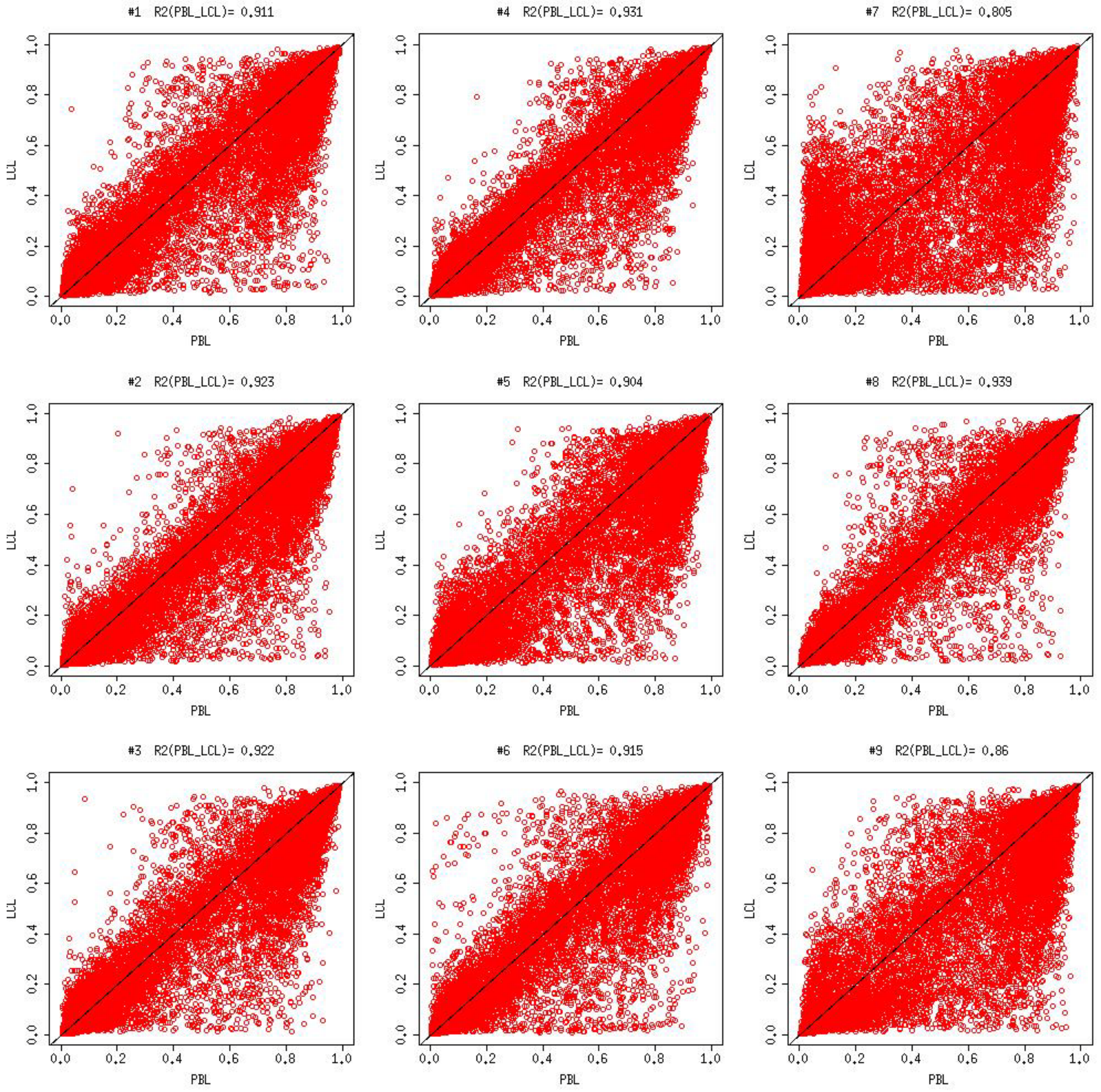

Supplementary S2a. Scatter plot of PBL and Artery of sample 1 to 9

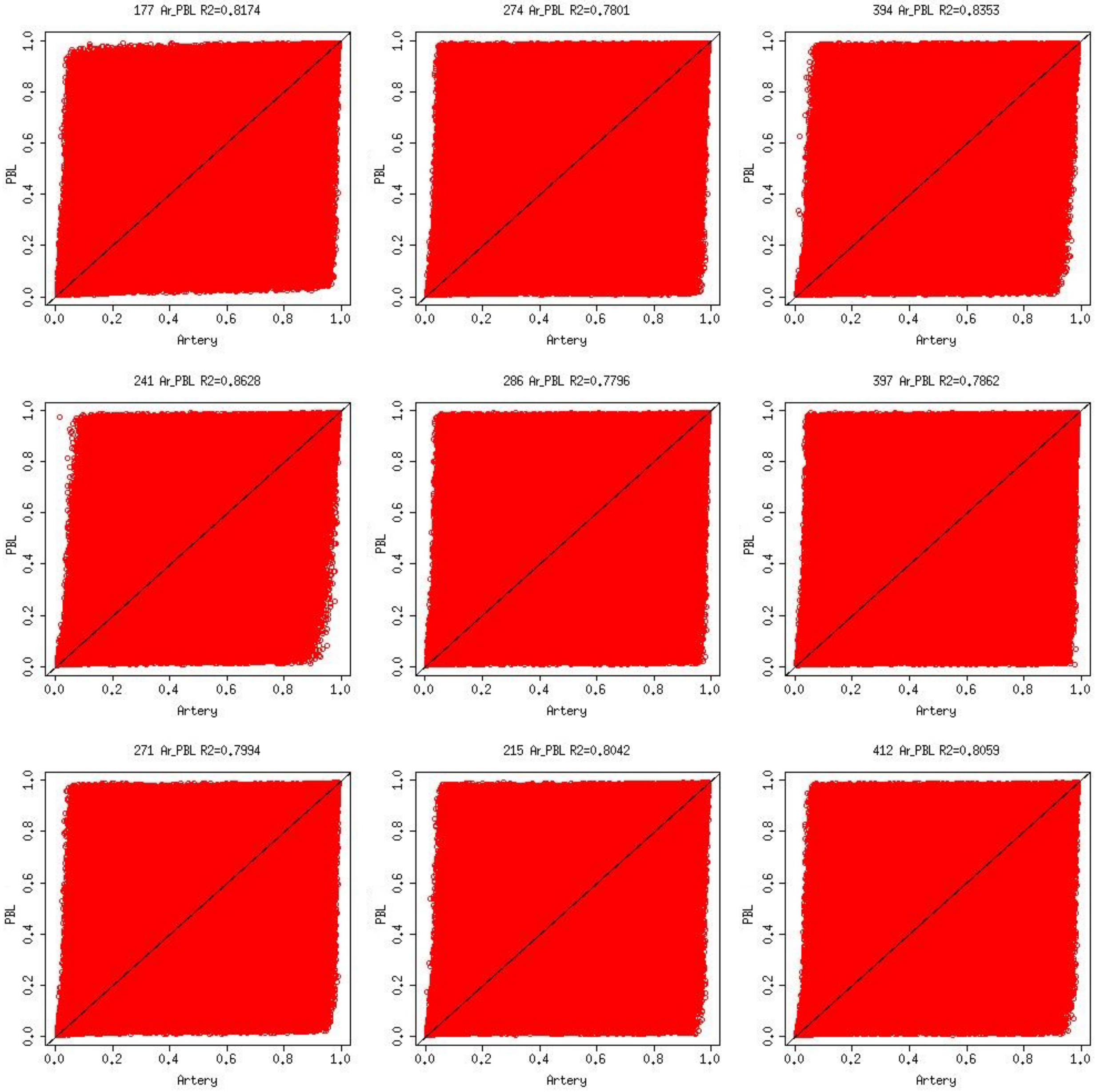

Supplementary S2b. Scatter plot of PBL and Atrium of sample 1 to 9

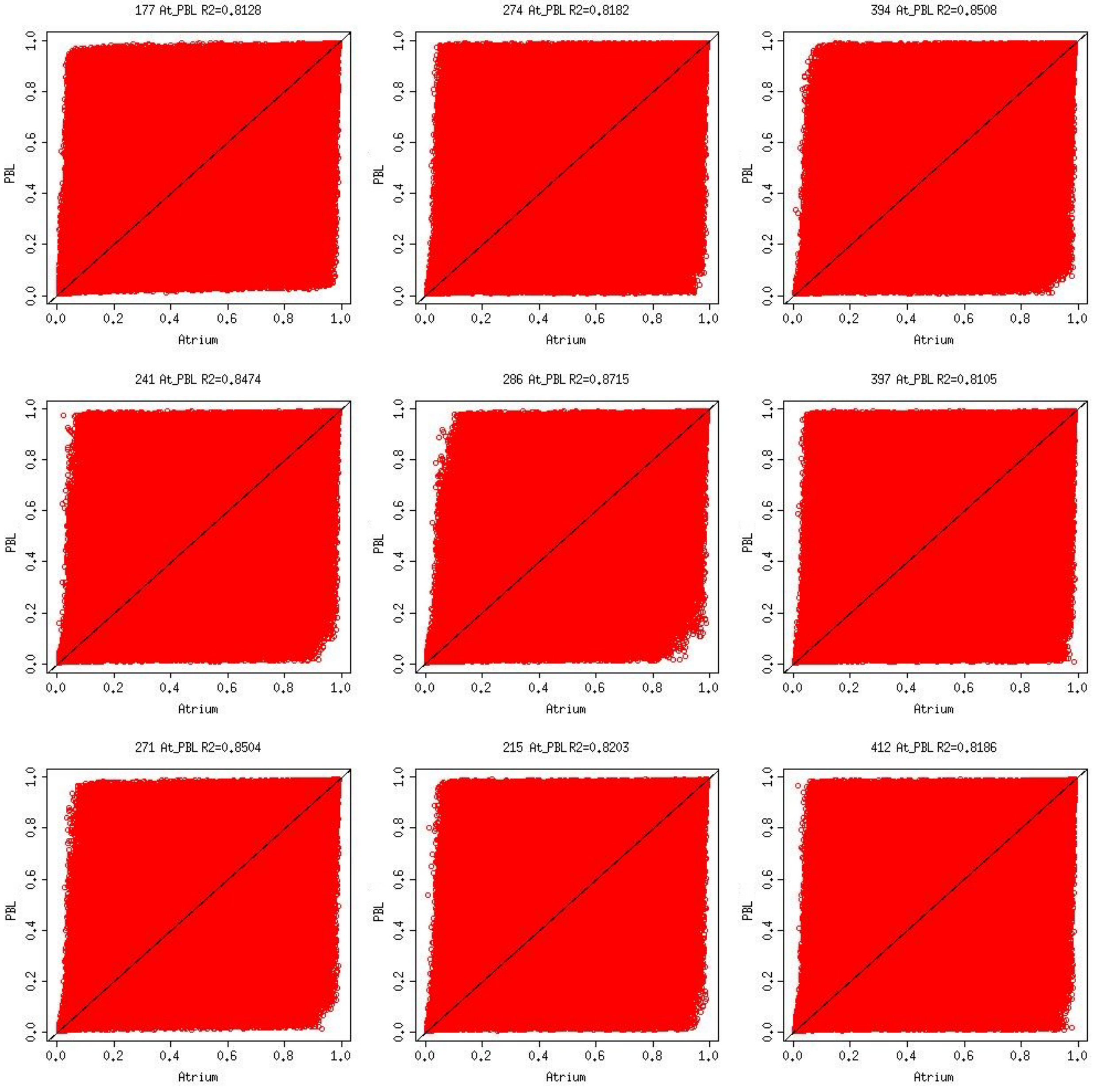

Supplementary S2c. Scatter plot of Atrium and Artery of sample 1 to 9

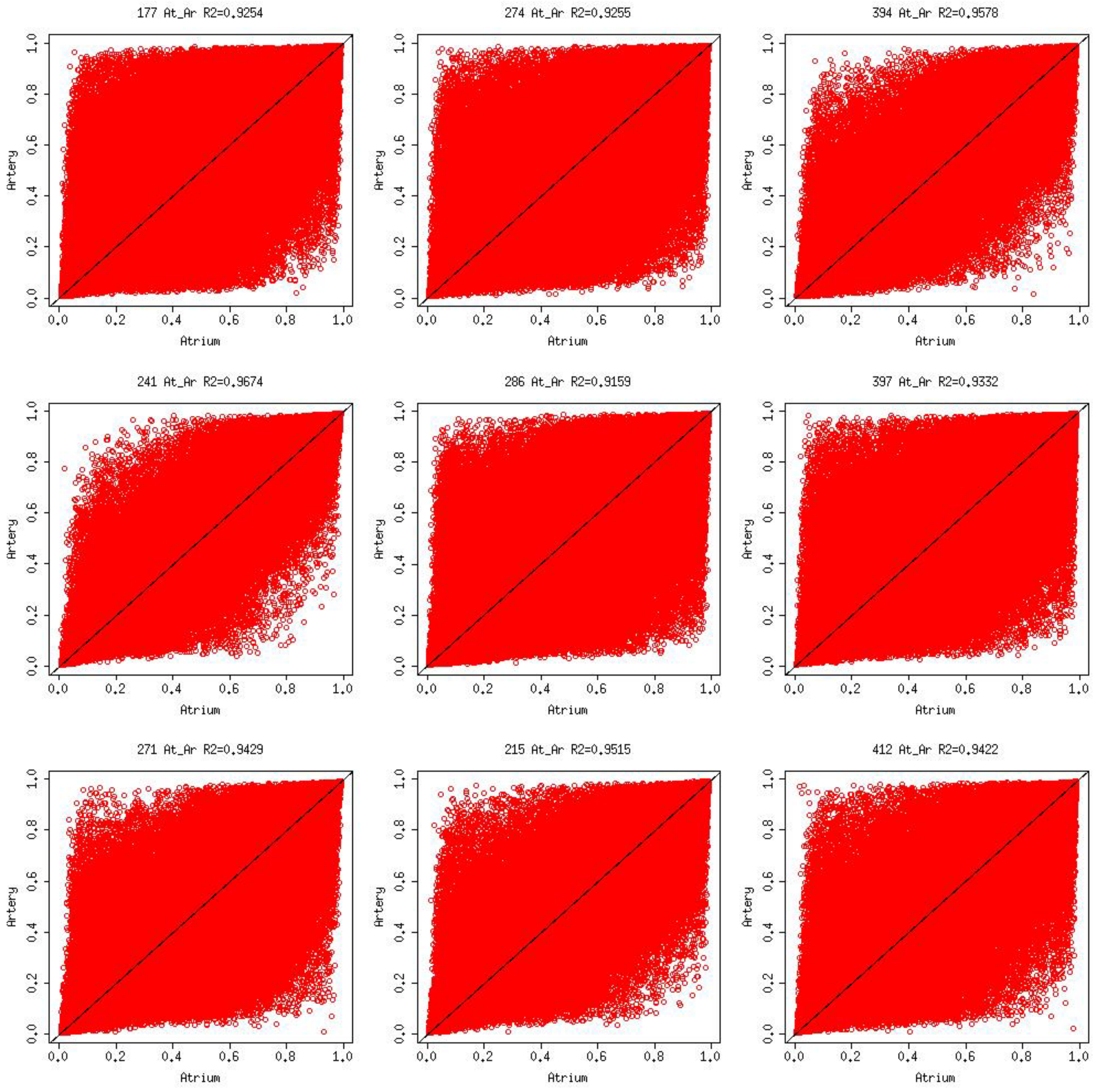

Supplementary S3. Scatter plot of PBL - LCL of sample 1 to 9

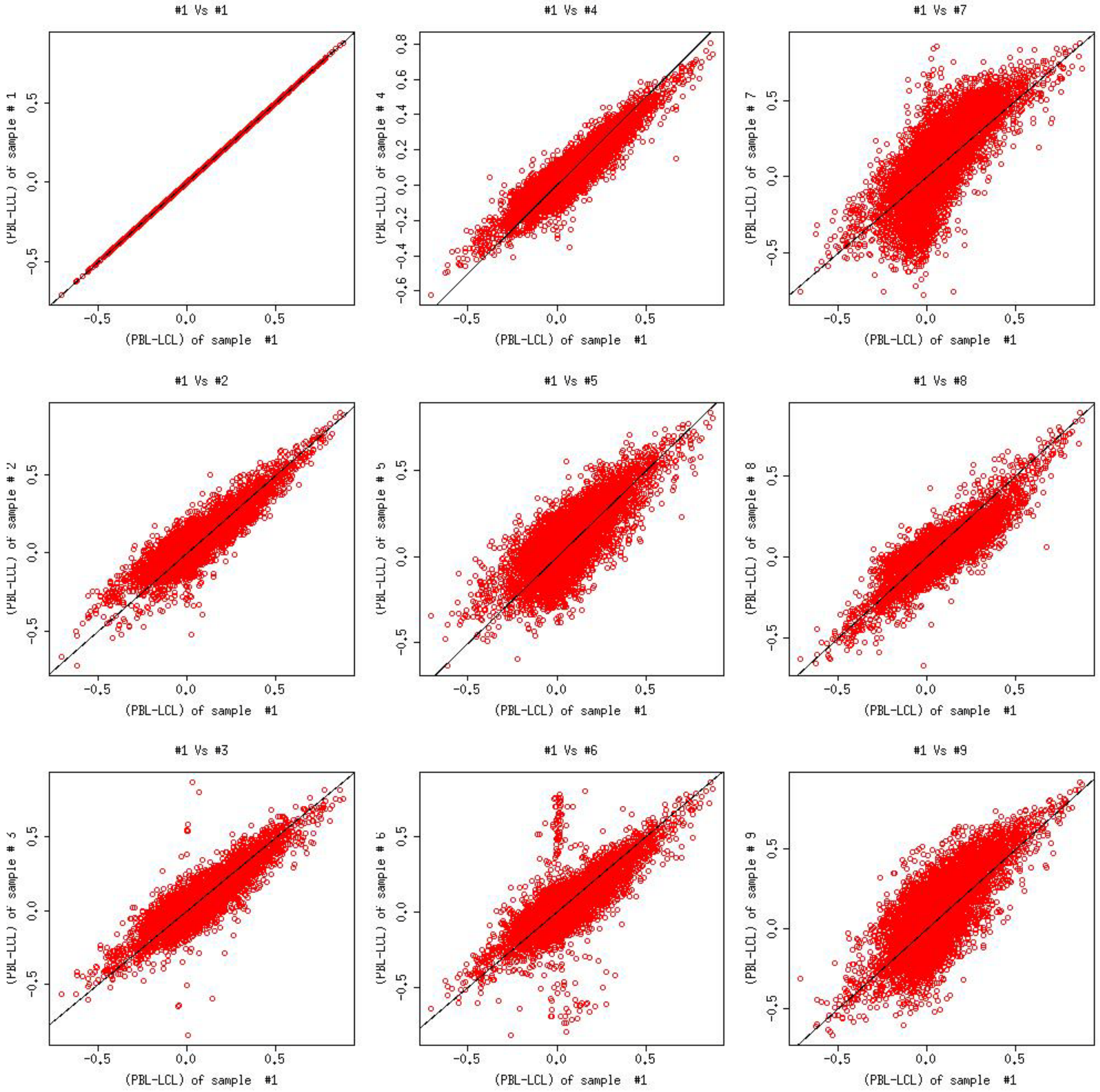

Supplementary S4a. Scatter plot of Artery - PBL of sample 1 to 9

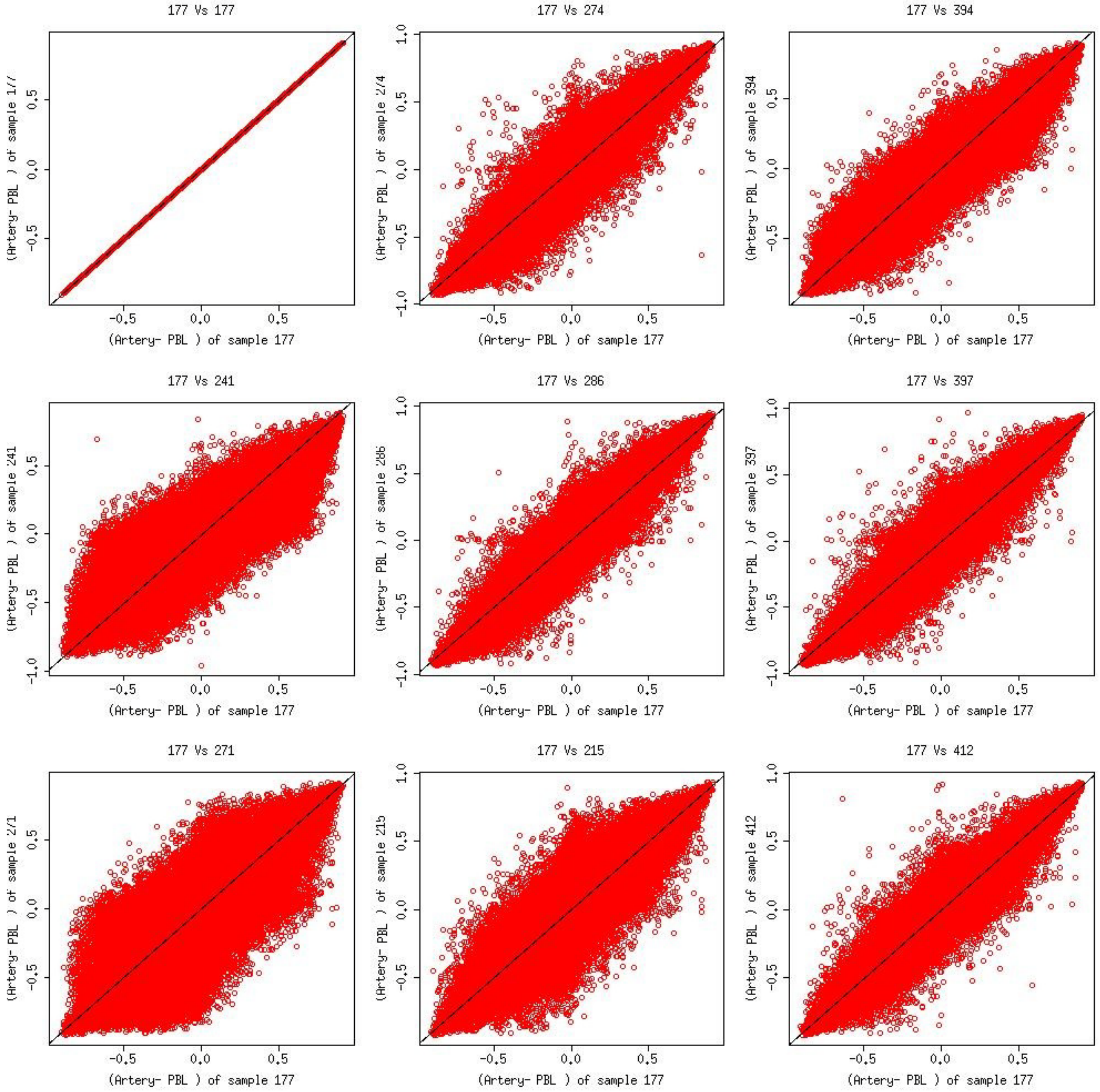

Supplementary S4b. Scatter plot of Atrium - PBL of sample 1 to 9

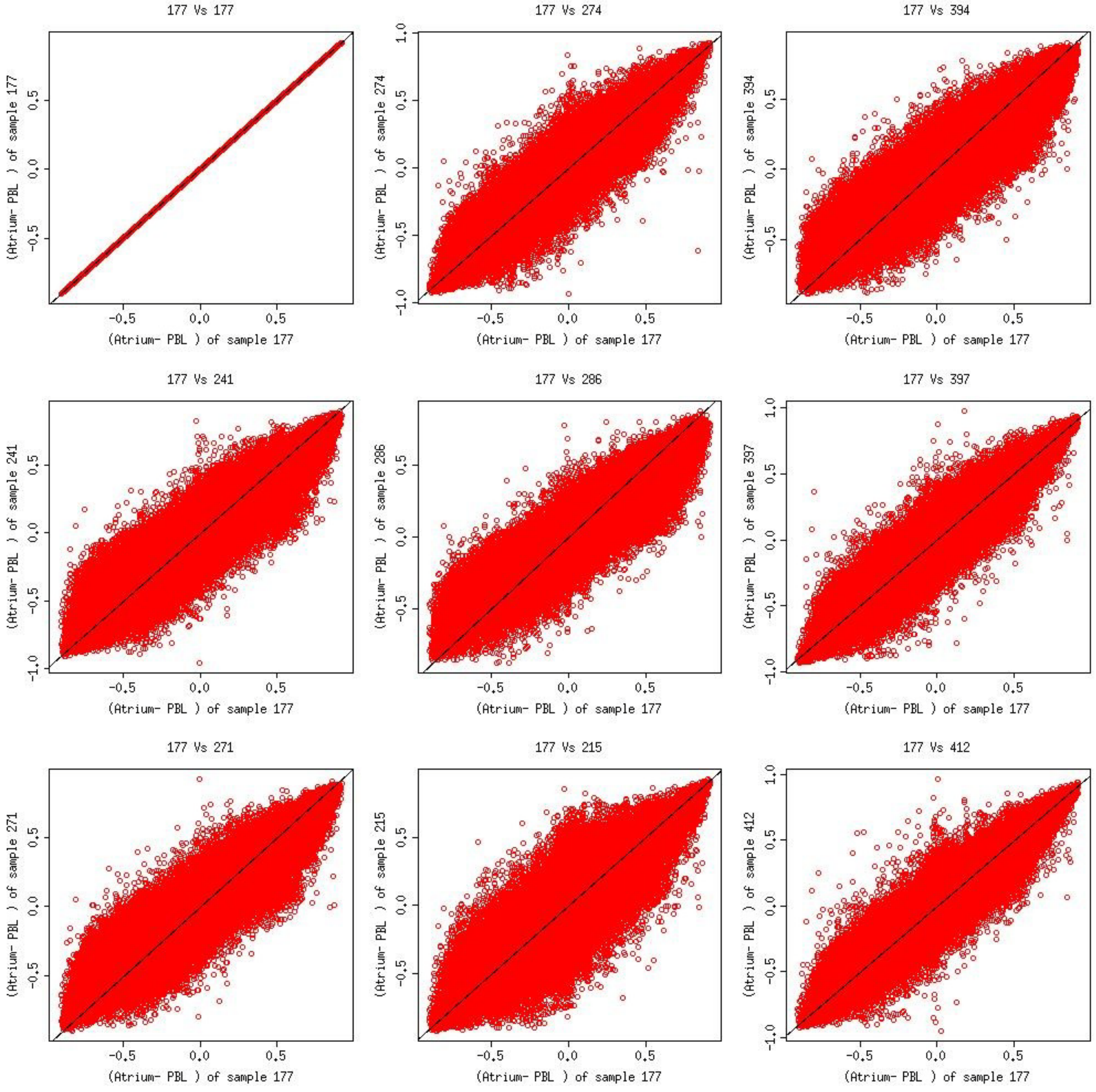

Supplementary S4c. Scatter plot of Atrium - Artery of sample 1 to 9

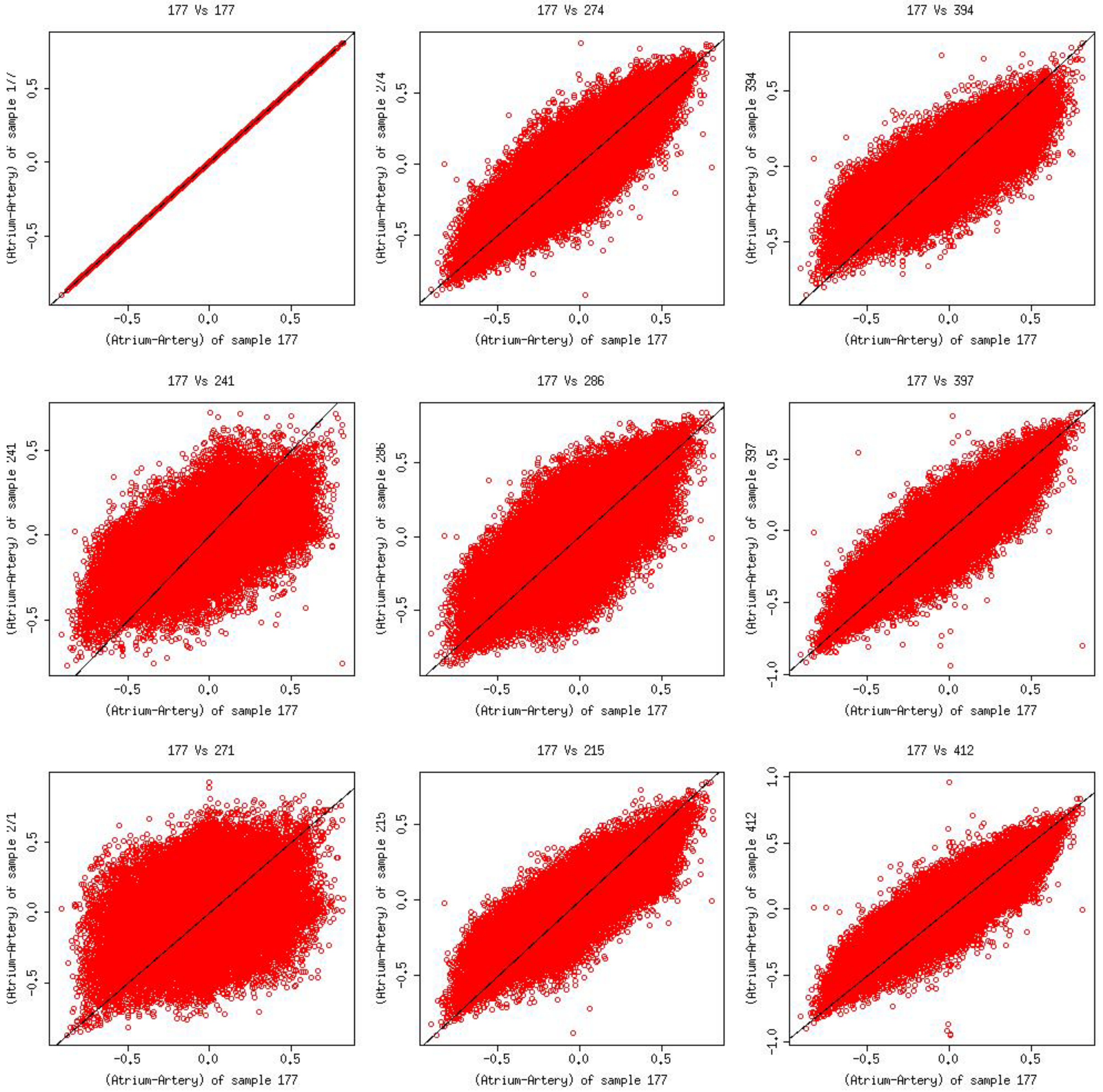

Supplementary S5a. Scatter plot of LCL, PBL and predicted PBL (LM) of sample 1 to 9

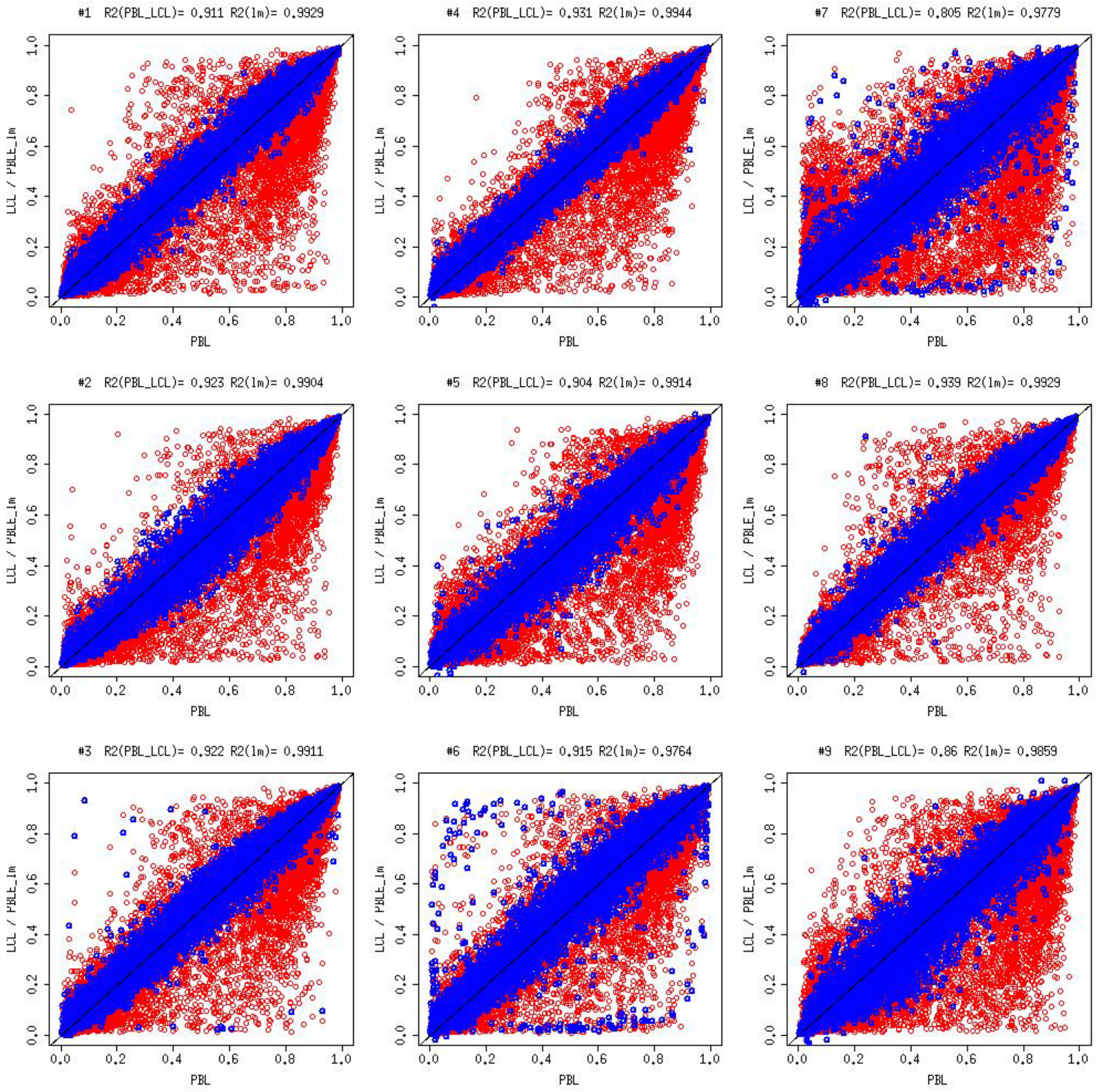

(The red circles represent LCL and PBL, the blue circles represent PBLE_lm and PBL, and PBLE_lm is the predicted PBL by using linear regression model)

Supplementary S5b. Scatter plot of LCL, PBL and predicted PBL (LM, SVM) of sample 1 to 9

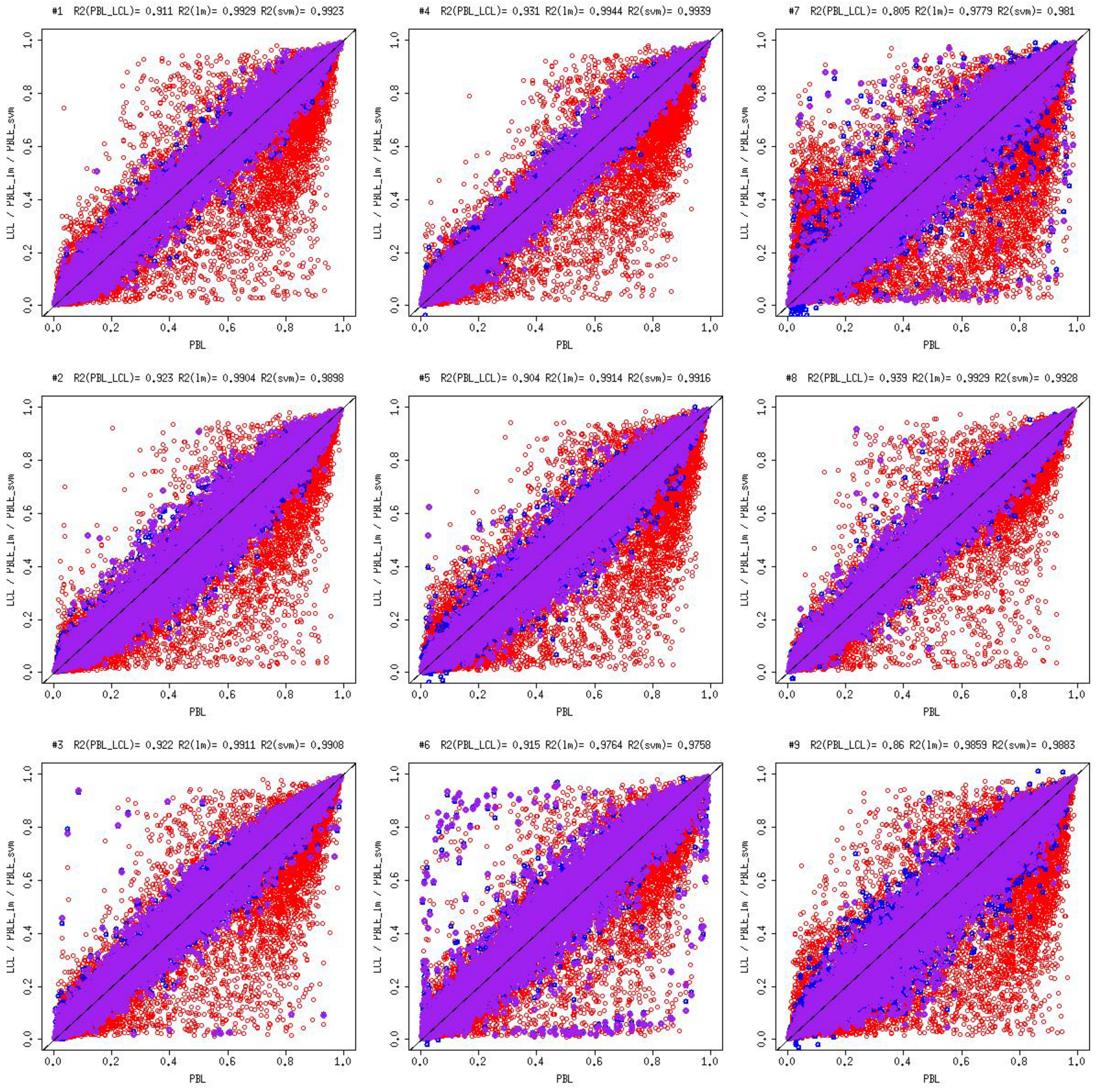

(The red circles represent LCL and PBL, the blue circles represent PBLE_lm and PBL, the purple circles represent PBLE_svm and PBL, PBLE_lm is the predicted PBL by using linear regression model, and PBLE_svm is the predicted PBL by using svm model)

Supplementary S5c. Scatter plot of PBL - LCL, PBL - predicted PBL (LM) of sample 1 to 9

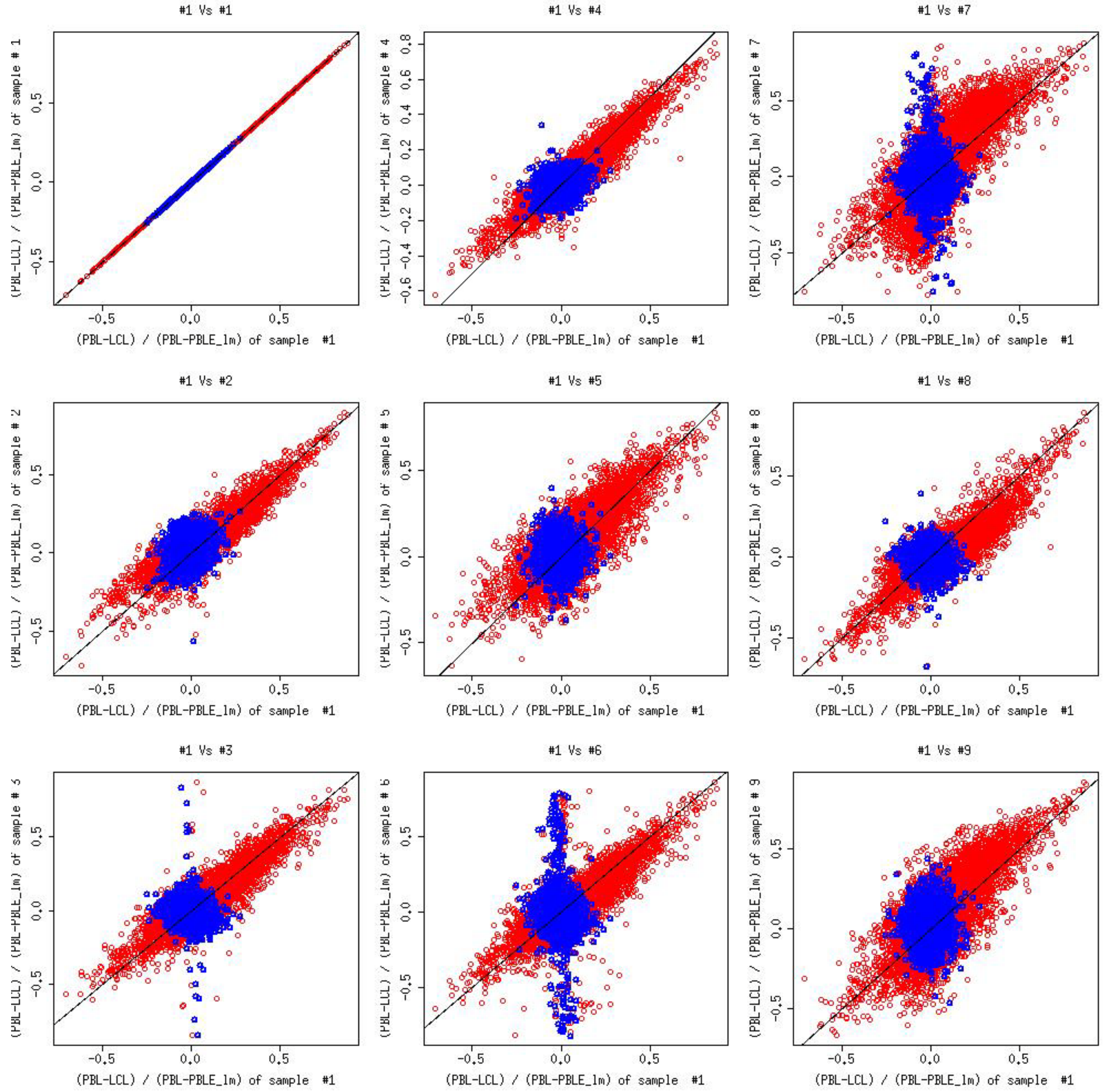

(The red circles represent difference of LCL and PBL, the blue circles represent difference of PBLE_lm and PBL, and PBLE_lm is the predicted PBL by using linear regression model)

Supplementary S5d. Scatter plot of PBL - LCL, PBL - predicted PBL (LM, SVM) of sample 1 to 9

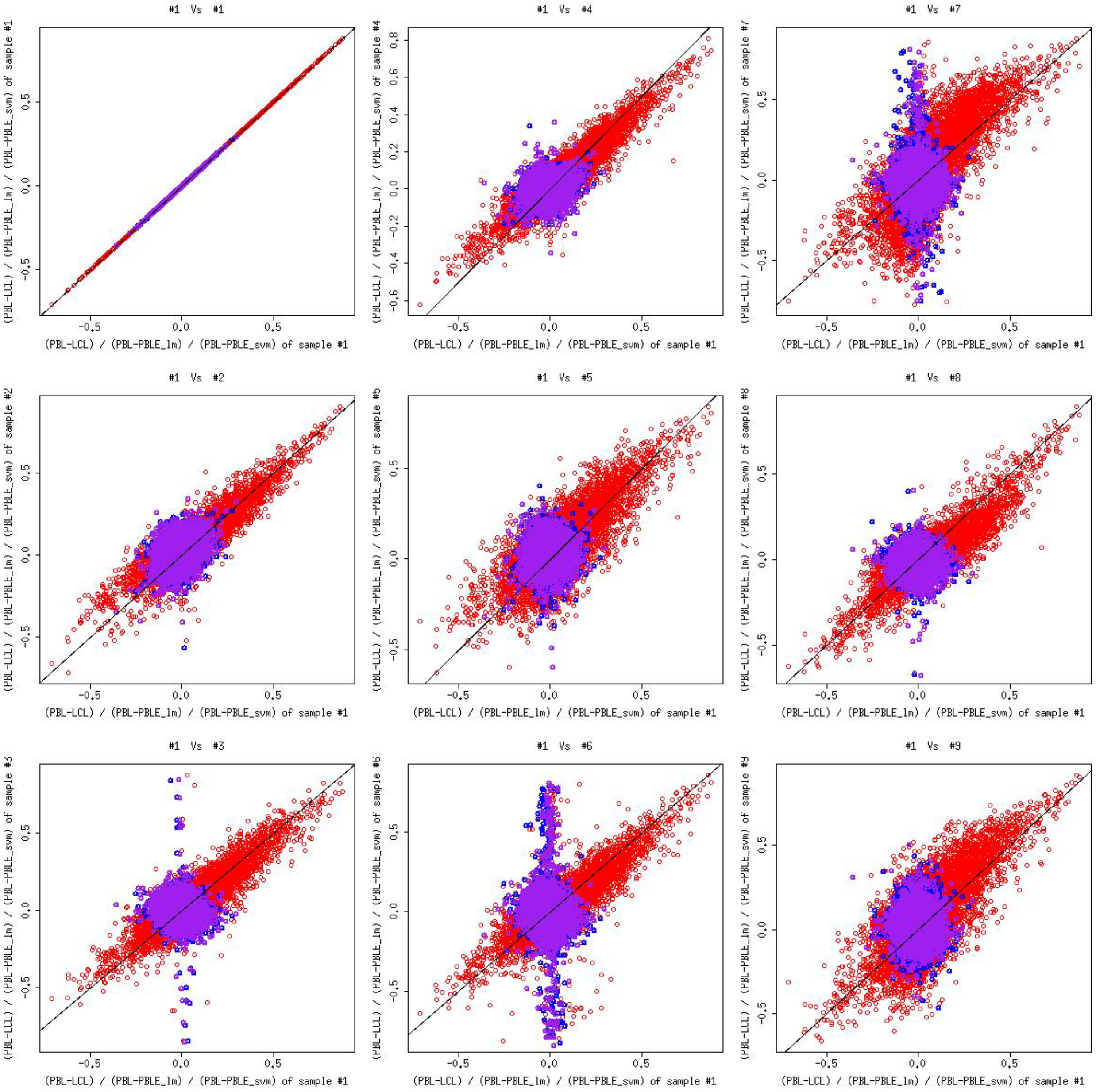

(The red circles represent the difference of LCL and PBL, the blue circles represent the difference of PBLE_lm and PBL, the purple circles represent the difference of PBLE_svm and PBL, PBLE_lm is the predicted PBL by using linear regression model, PBLE_svm is the predicted PBL by using svm model)

Supplementary S6a. Scatter plot of PBL, Artery and predicted Artery (LM) of sample 1 to 9

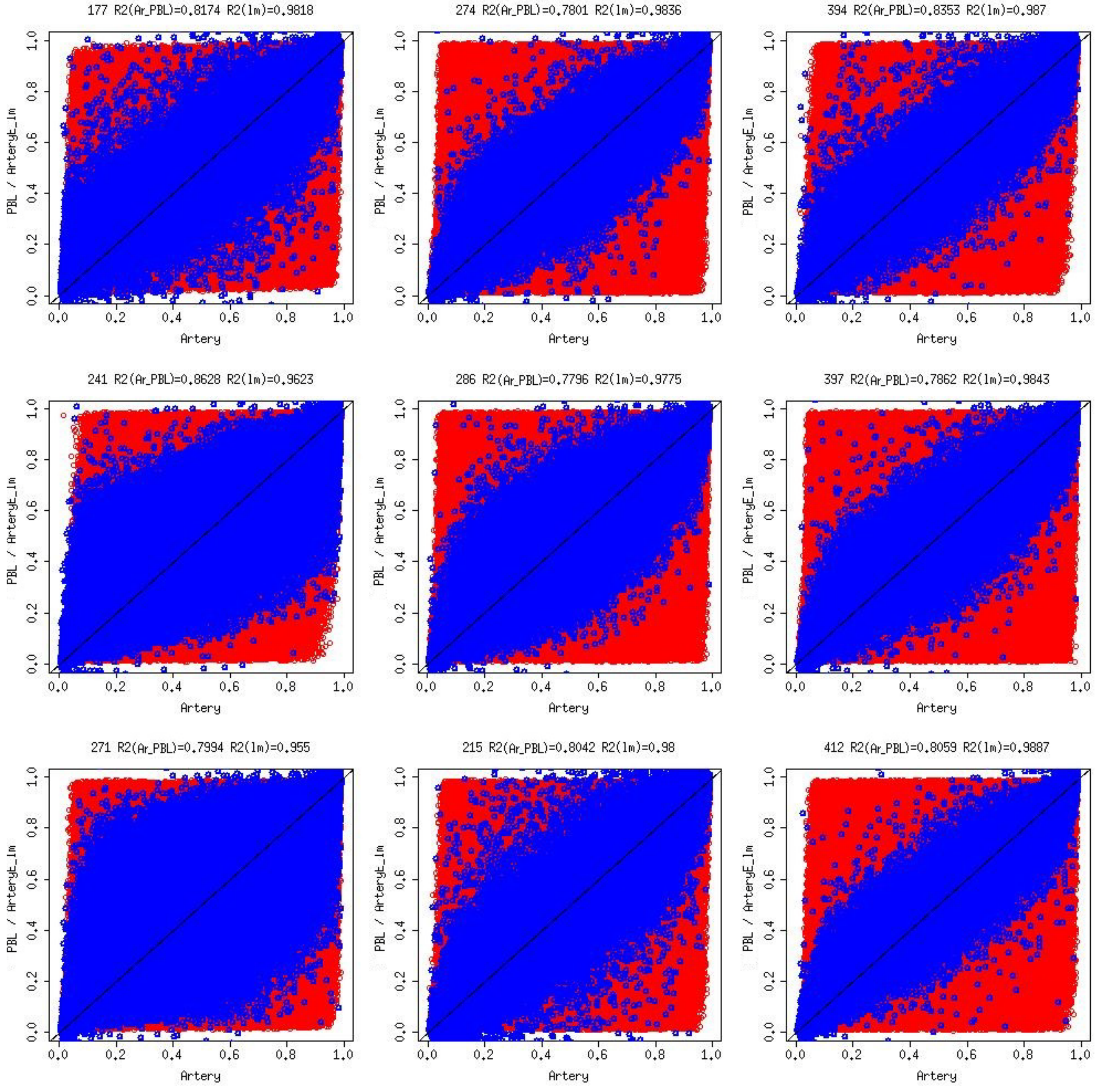

(The red circles represent PBL and Artery, the blue circles represent ArteryE_lm and Artery, ArteryE_lm is the predicted Artery by using linear regression model)

Supplementary S6b. Scatter plot of PBL, Artery and predicted Artery (LM, SVM) of sample 1 to 9

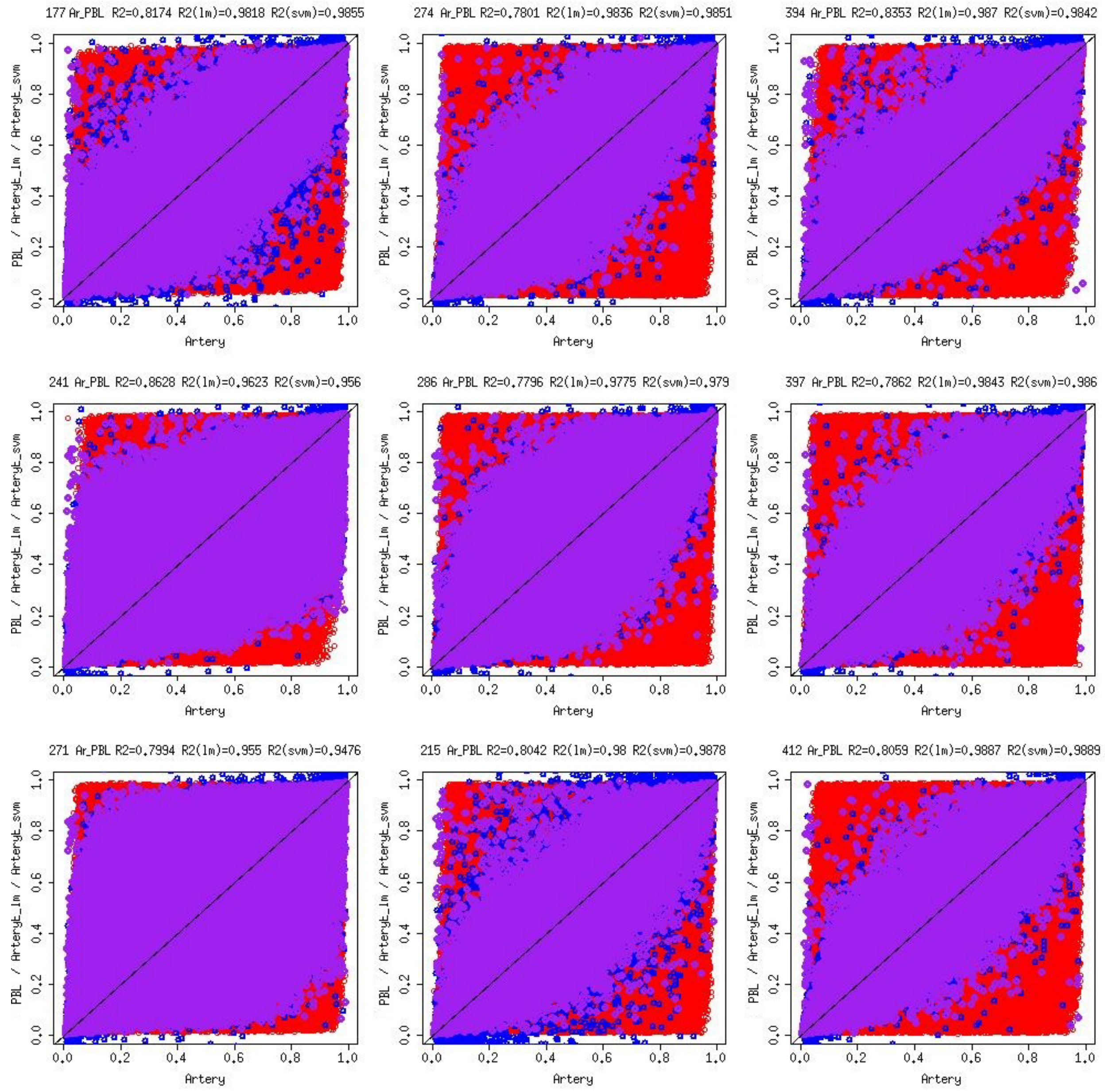

(The red circles represent PBL and Artery, the blue circles represent ArteryE_lm and Artery, the purple circles represent ArteryE_svm and Artery, ArteryE_lm is the predicted Artery by using linear regression model, ArteryE_svm is the predicted Artery by using svm model)

Supplementary S6c. Scatter plot of Artery - PBL, Artery - predicted Artery (LM) of sample 1 to 9

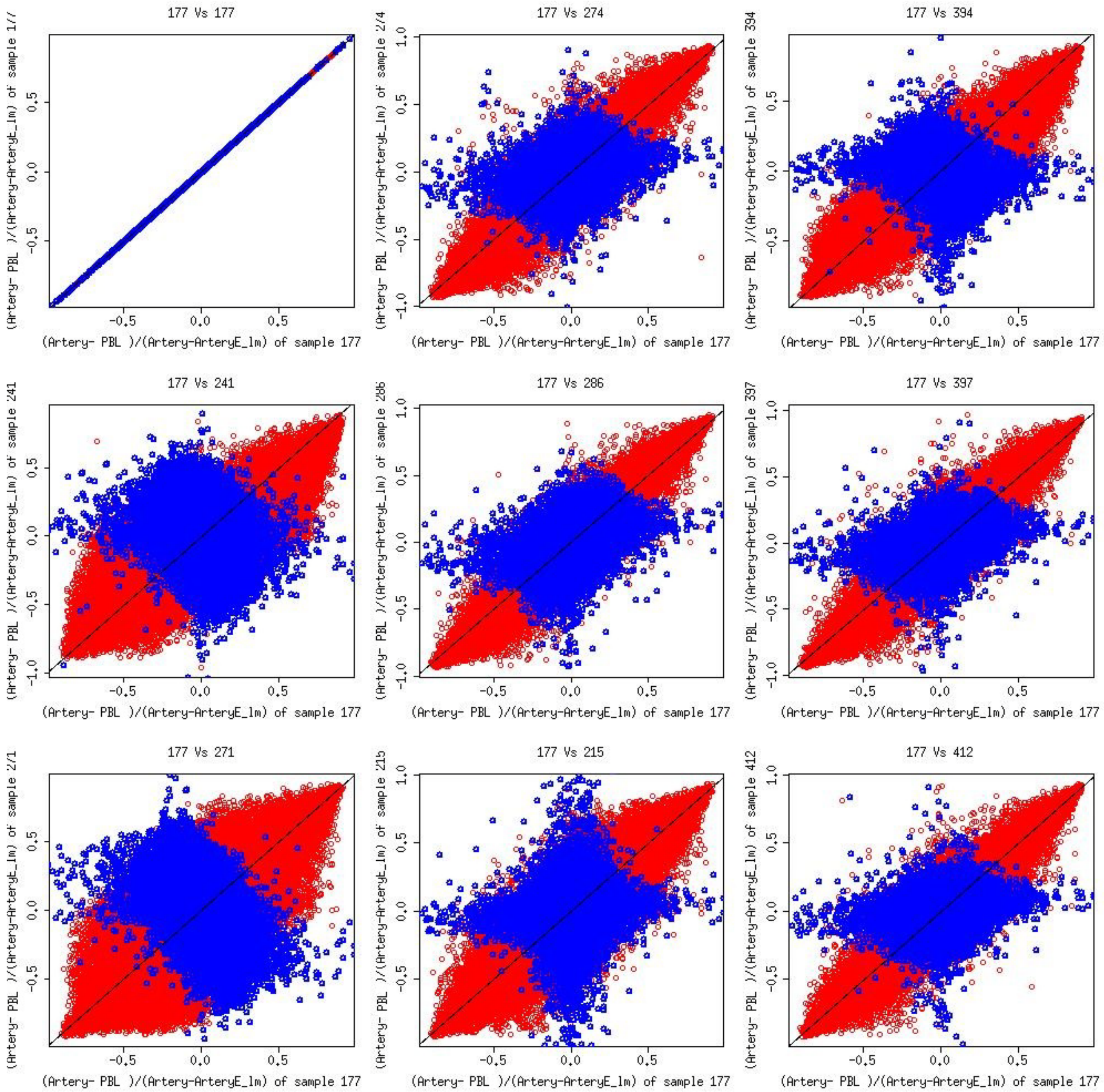

(The red circles represent the difference of PBL and Artery, the blue circles represent the difference of ArteryE_lm and Artery, ArteryE_lm is the predicted Artery by using linear regression model)

Supplementary S6d. Scatter plot of Artery - PBL, Artery - predicted Artery (LM, SVM) of sample 1 to 9

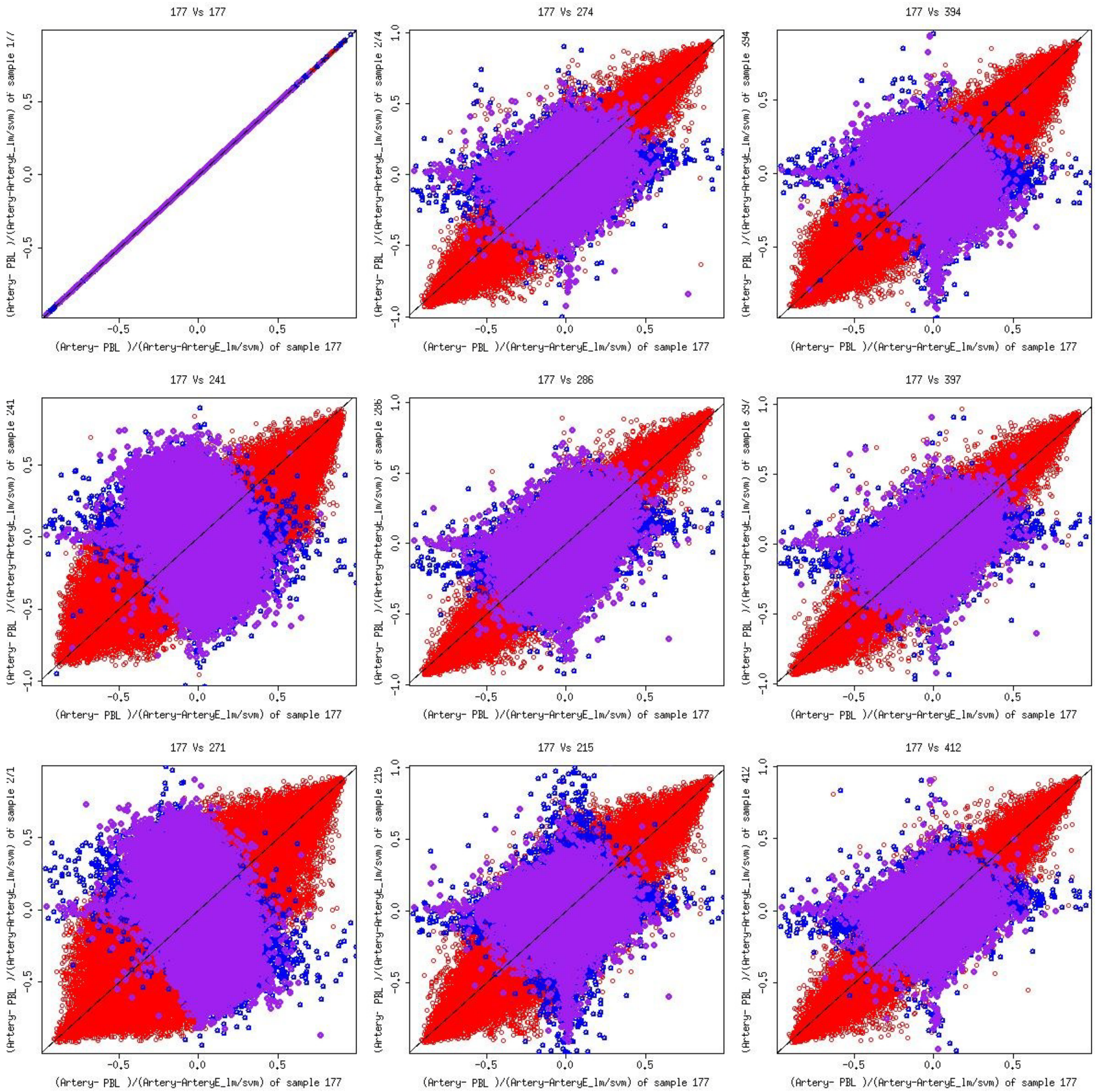

(The red circles represent the difference of PBL and Artery, the blue circles represent the difference of ArteryE_lm and Artery, the purple circles represent the difference of ArteryE_svm and Artery, ArteryE_lm is the predicted Artery by using linear regression model, ArteryE_svm is the predicted Artery by using svm model)

Supplementary S7a. Scatter plot of PBL, Atrium and predicted Atrium (LM) of sample 1 to 9

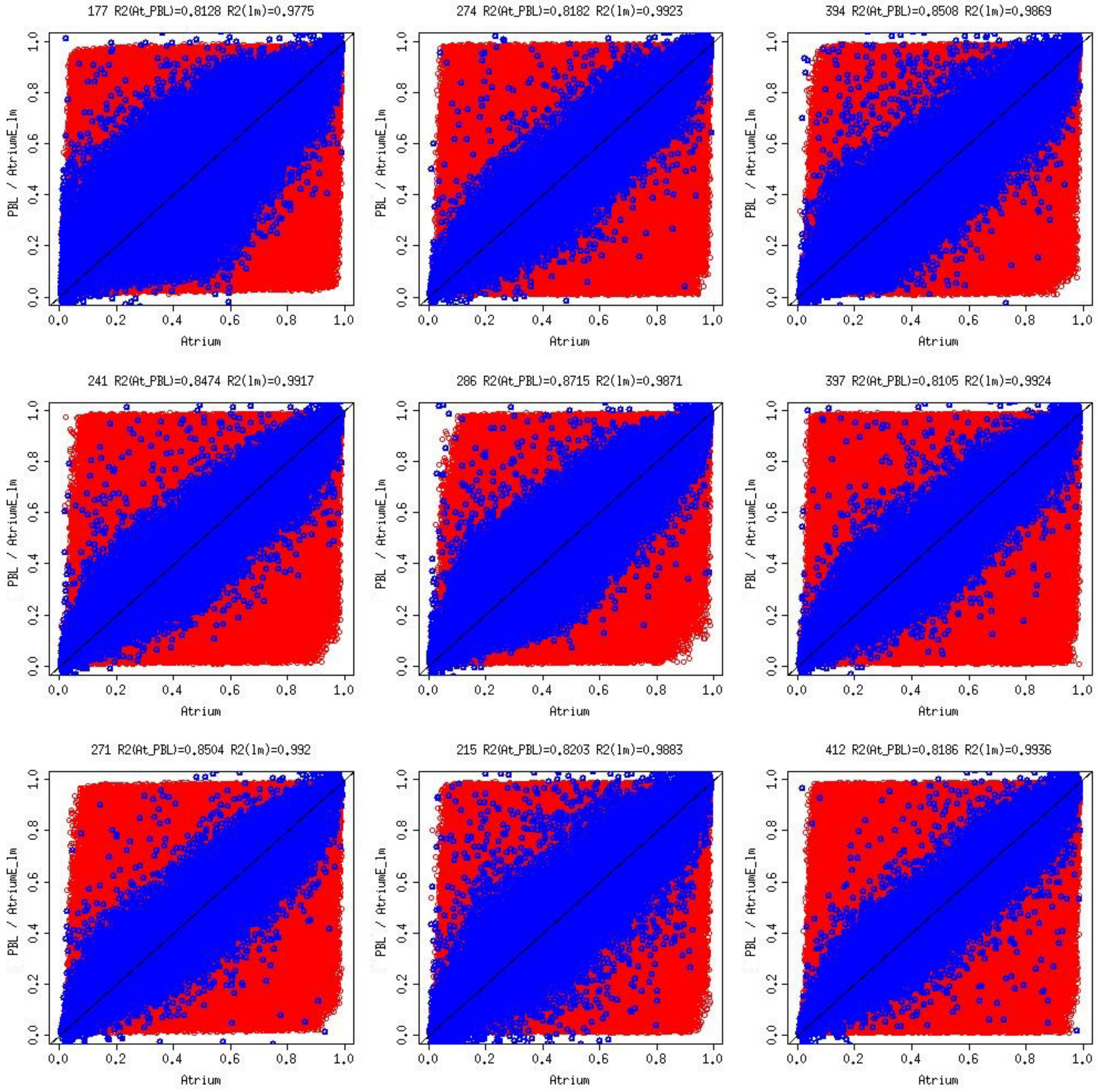

(The red circles represent PBL and Atrium, the blue circles represent AtriumE_lm and Atrium, AtriumE_lm is the predicted Atrium by using linear regression model)

Supplementary S7b. Scatter plot of PBL, Atrium and predicted Atrium (LM, SVM) of sample 1 to 9

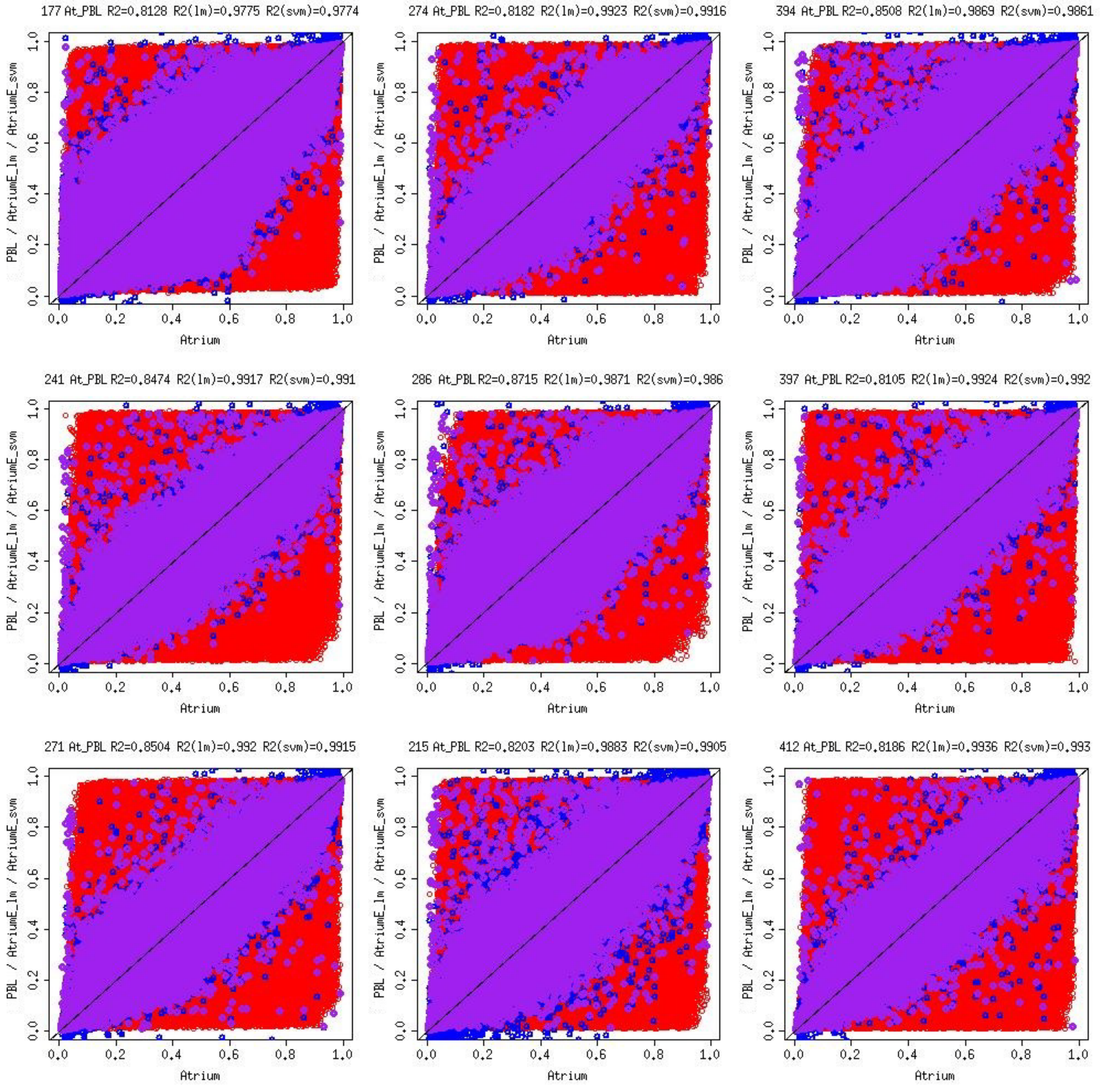

(The red circles represent PBL and Atrium, the blue circles represent AtriumE_lm and Atrium, the purple circles represent AtriumE_svm and Atrium, AtriumE_lm is the predicted Atrium by using linear regression model, AtriumE_svm is the predicted Atrium by using svm model)

Supplementary S7c. Scatter plot of Atrium - PBL, Atrium - predicted Atrium (LM) of sample 1 to 9

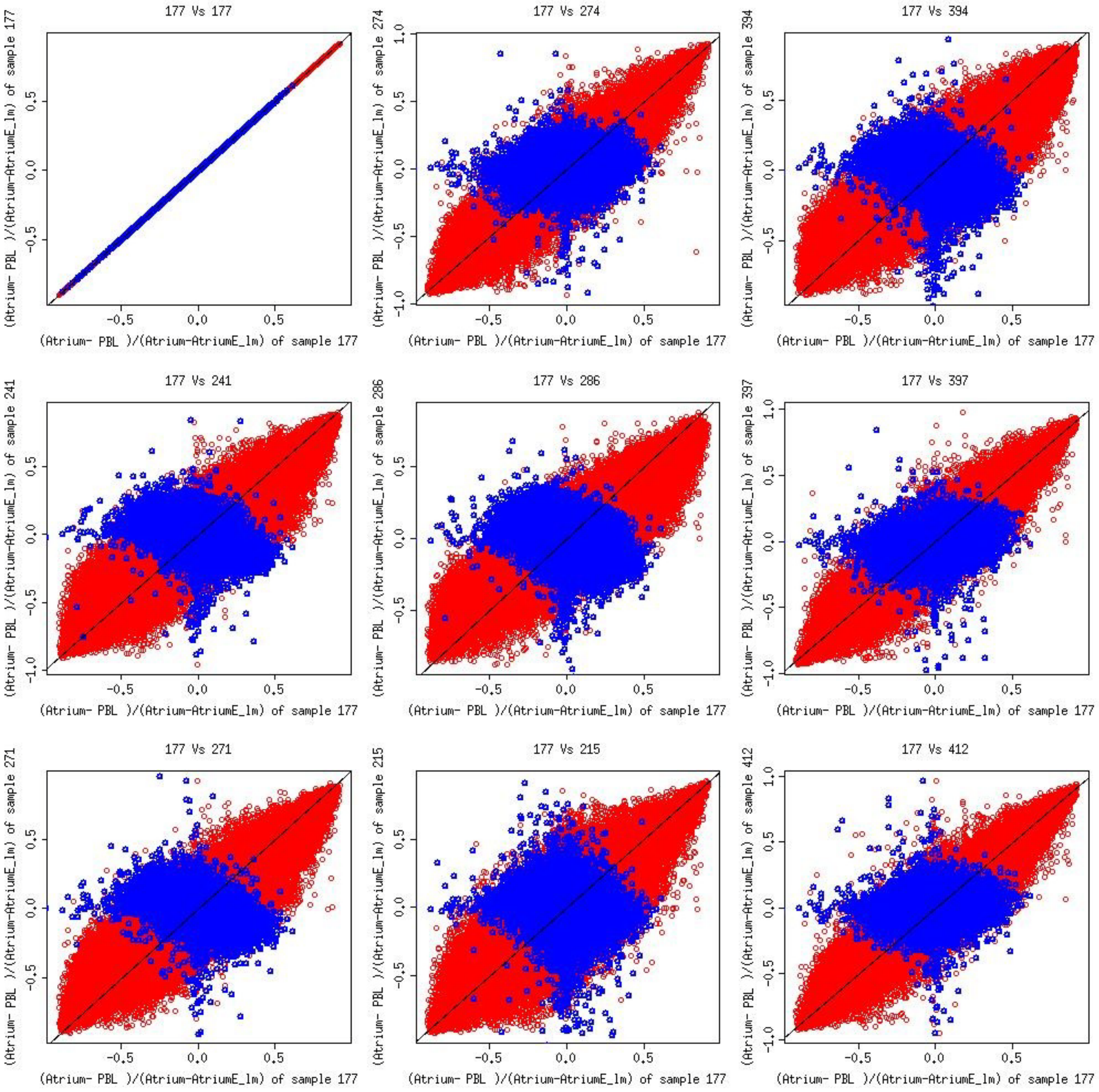

(The red circles represent the difference of PBL and Atrium, the blue circles represent the difference of AtriumE_lm and Artery, AtriumE_lm is the predicted Atrium by using linear regression model)

Supplementary S7d. Scatter plot of Atrium - PBL, Atrium - predicted Atrium (LM, SVM) of sample 1 to 9

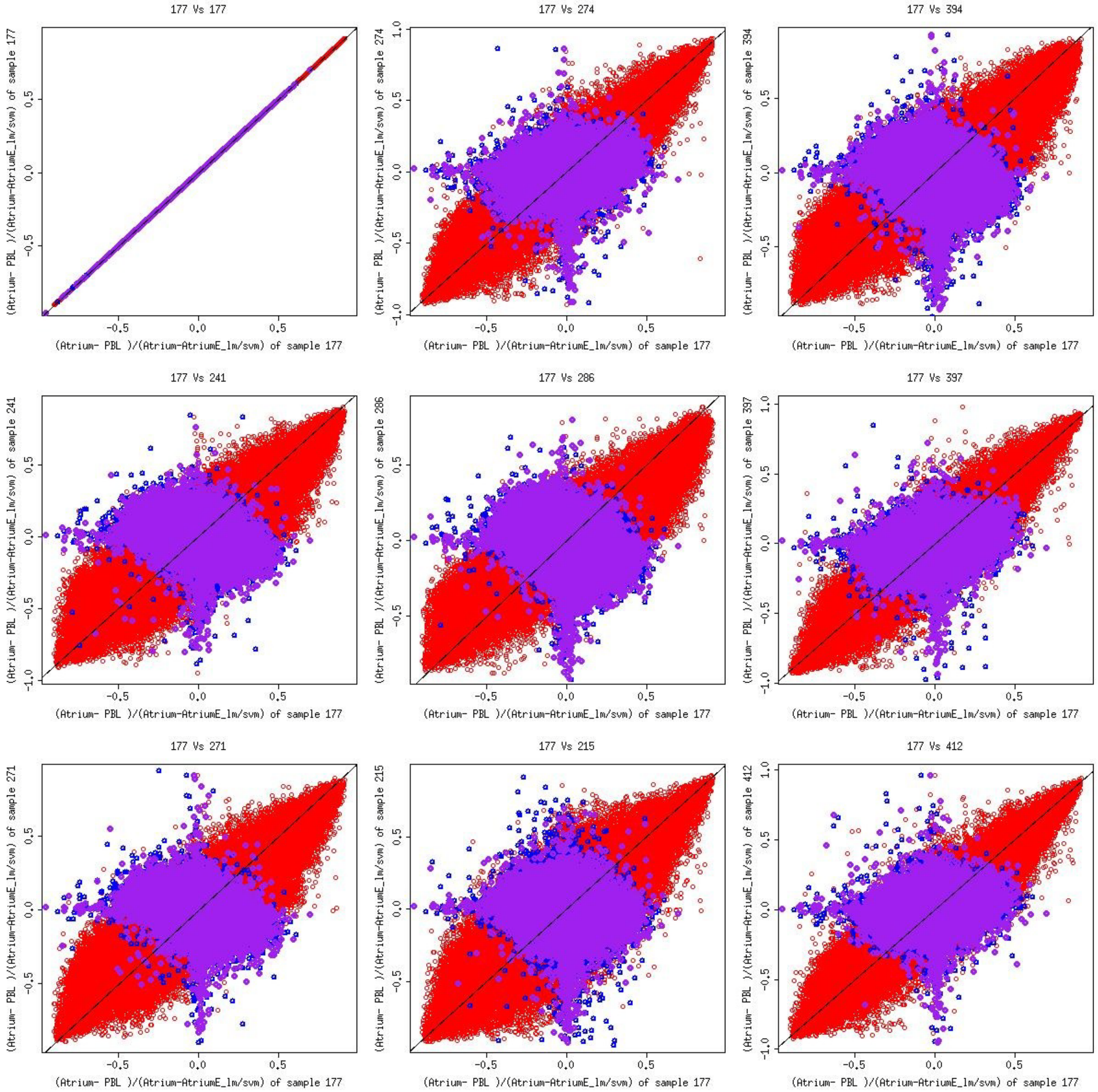

(The red circles represent the difference of PBL and Atrium, the blue circles represent the difference of AtriumE_lm and Artery, the purple circles represent the difference of AtriumE_svm and Artery, AtriumE_lm is the predicted Atrium by using linear regression model, AtriumE_svm is the predicted Atrium by using svm model)

Supplementary S8. Methylation pattern across tissues and between-tissue difference across individuals

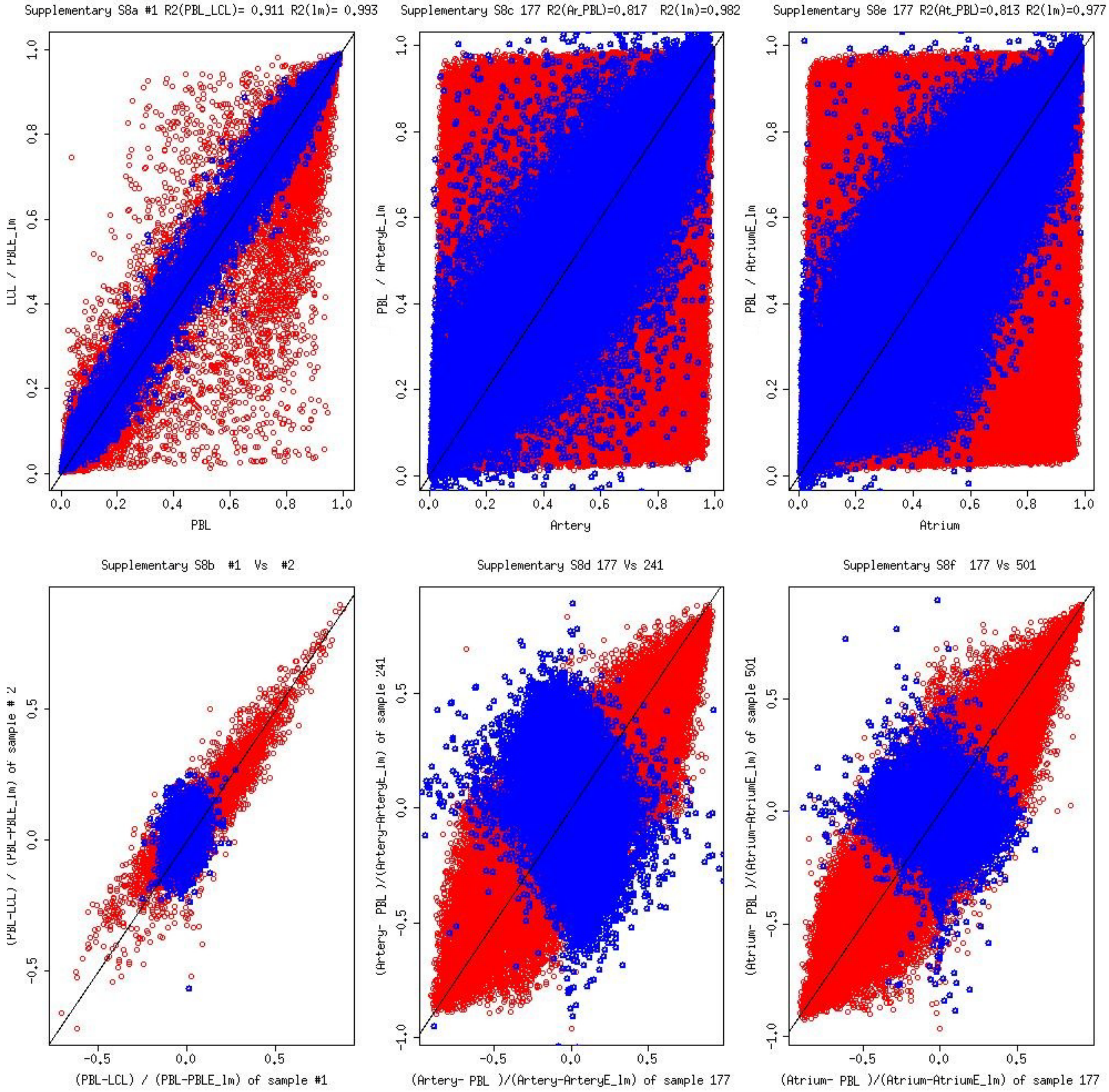

Supplementary S8a. Scatter plot of PBL vs. LCL (red circles) and PBL vs. LM predicted PBL (blue circles) of sample #1

Supplementary S8b. Scatter plot of PBL – LCL in sample #1 vs. PBL – LCL in sample #2 (red circles) and PBL – LM predicted PBL of sample #1 vs. PBL – LM predicted PBL in sample #2 (blue circles)

Supplementary S8c. Scatter plot of Artery vs. PBL (red circles) and Artery vs. LM predicted Artery (blue circles) of sample 177

Supplementary S8d. Scatter plot of Artery – PBL in sample 177 vs. Artery – PBL in sample 241 (red circles) and Artery – LM predicted Artery of sample 177 vs. Artery – LM predicted Artery in sample 241 (blue circles)

Supplementary S8e. Scatter plot of Atrium vs. PBL (red circles) and Atrium vs. LM predicted Atrium (blue circles) of sample 177

Supplementary S8f. Scatter plot of Atrium – PBL in sample 177 vs. Atrium – PBL in sample 501 (red circles) and Atrium – LM predicted Atrium of sample 177 vs. Atrium – LM predicted Atrium in sample 501 (blue circles)

* For scatter plots for all other samples, please refer to Supplementary Figures S5 and W-S5 for LCL-PBL, S6 and W-S6 for PBL-Artery, S7and W-S7 for PBL-Atrium.

Supplementary S9. Probe specific prediction accuracy based on LM model by methylation variation within target tissues

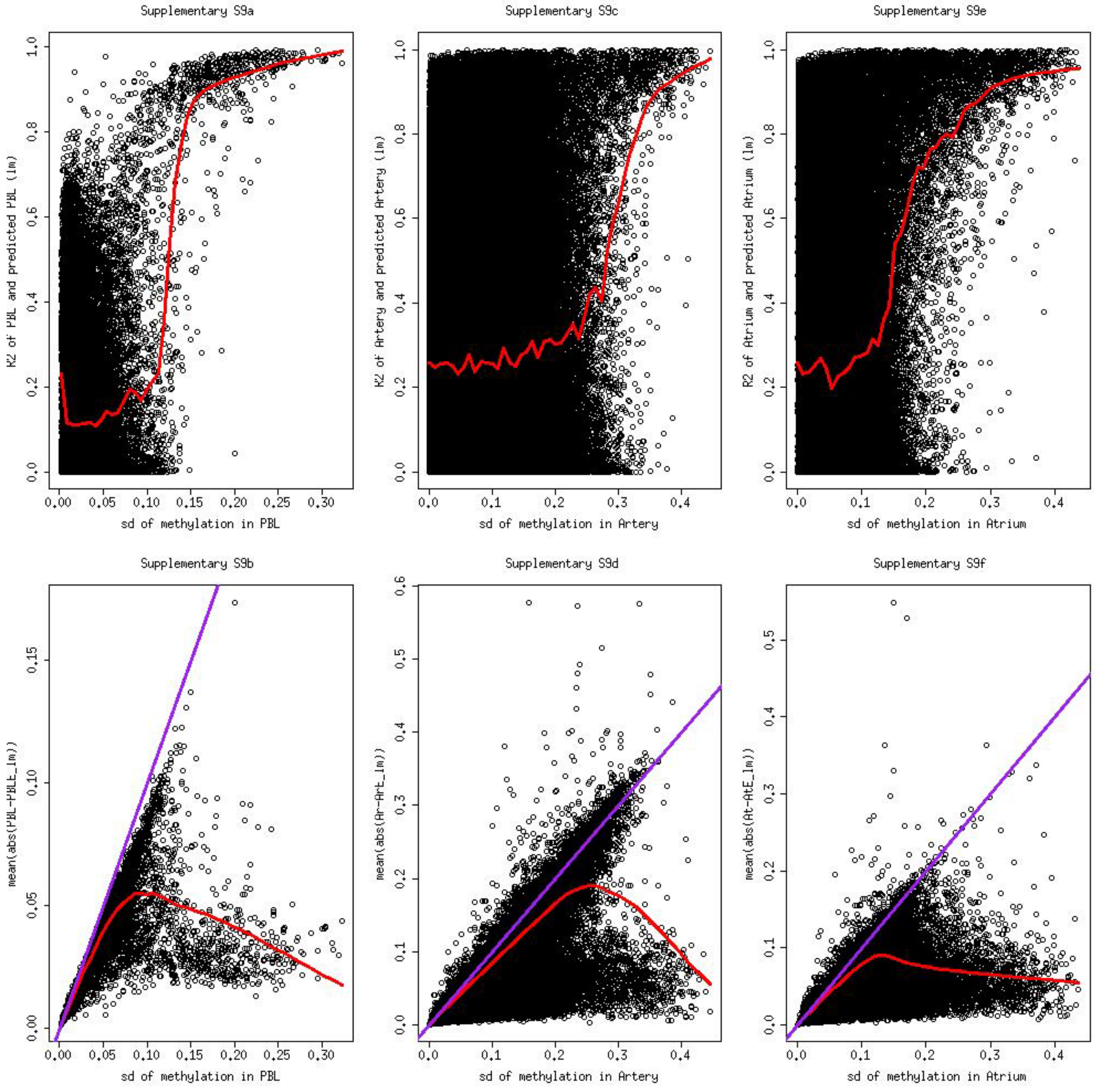

Supplementary S9a. Standard deviation (sd) of methylation in PBL vs. R^2 between PBL and predicted PBL based on LM

Supplementary S9b. Standard deviation (sd) of methylation in PBL vs. mean absolute value of difference between PBL and predicted PBL based on LM

Supplementary S9c. Standard deviation (sd) of methylation in Artery vs. R^2 between Artery and predicted Artery based on LM

Supplementary S9d. Standard deviation (sd) of methylation in Artery vs. mean absolute value of difference between Artery and predicted Artery based on LM

Supplementary S9e. Standard deviation (sd) of methylation in Atrium vs. R^2 between Atrium and predicted Atrium based on LM

Supplementary S9f. Standard deviation (sd) of methylation in Atrium vs. mean absolute value of difference between Atrium and predicted Atrium based on LM

* Each dot represents one probe on the Illumina array. The curve represents the LOESS smoothing average curve. The straight line in panel (b), (d) and (f) is the x=y line.

Supplementary S10. Density of predicted methylation level by true methylation in Artery for sample 271

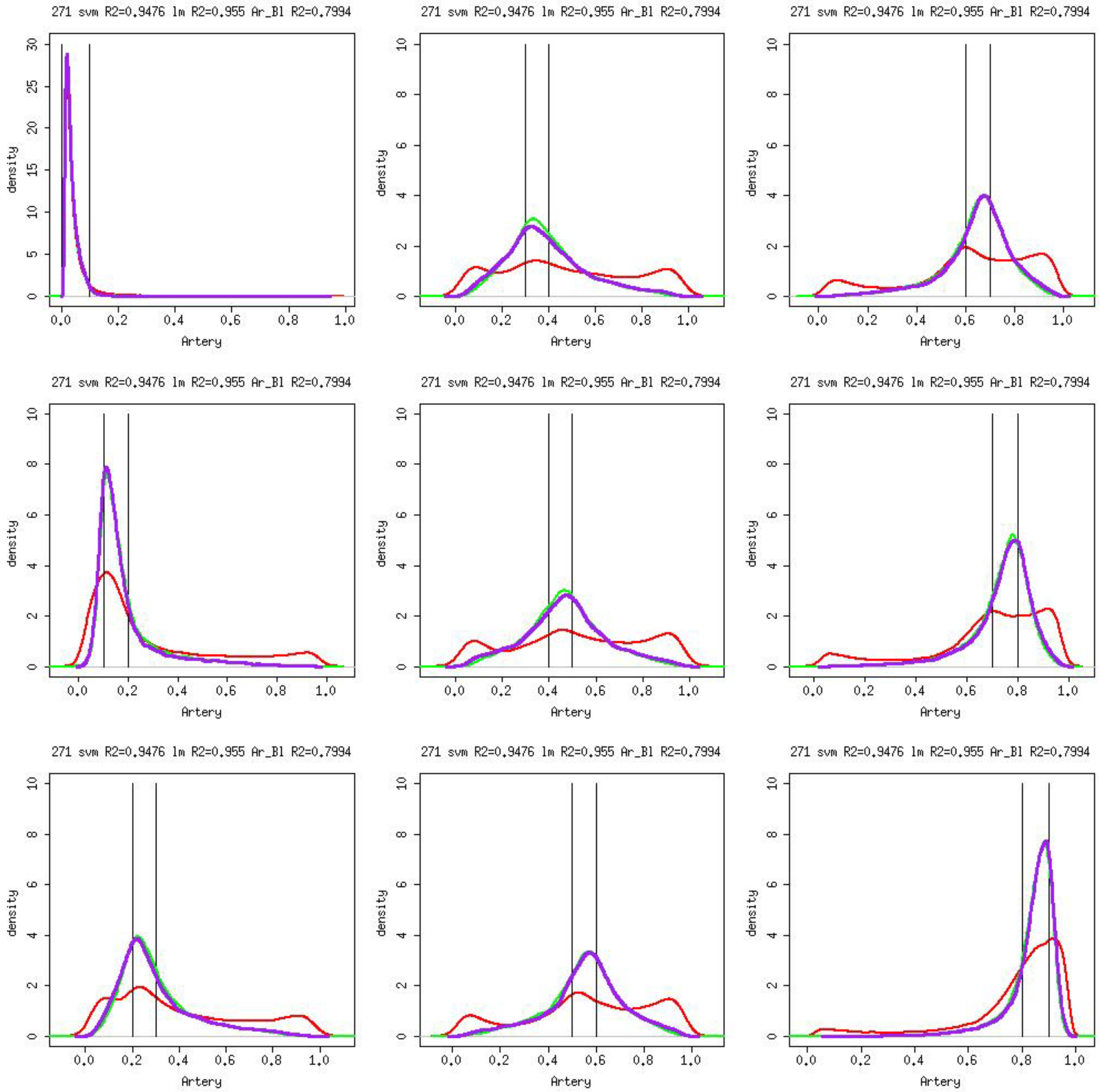

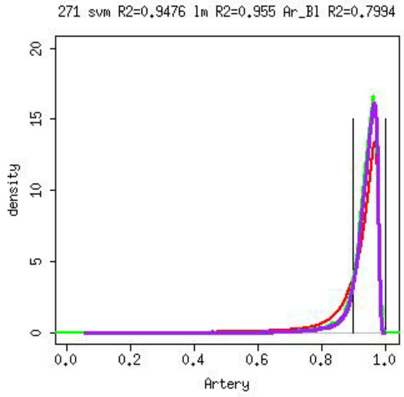

* Red line represents the density of methylation in PBL. Green line represents the density of the predicted artery methylation by using linear regression model. Purple line represents predicted methylation using SVM model. The two vertical lines represent the range of true methylation level in artery

Supplementary S11. Scatter plot of PBL, Artery and predicted Artery (LM, SVM) of sample 1 to 9

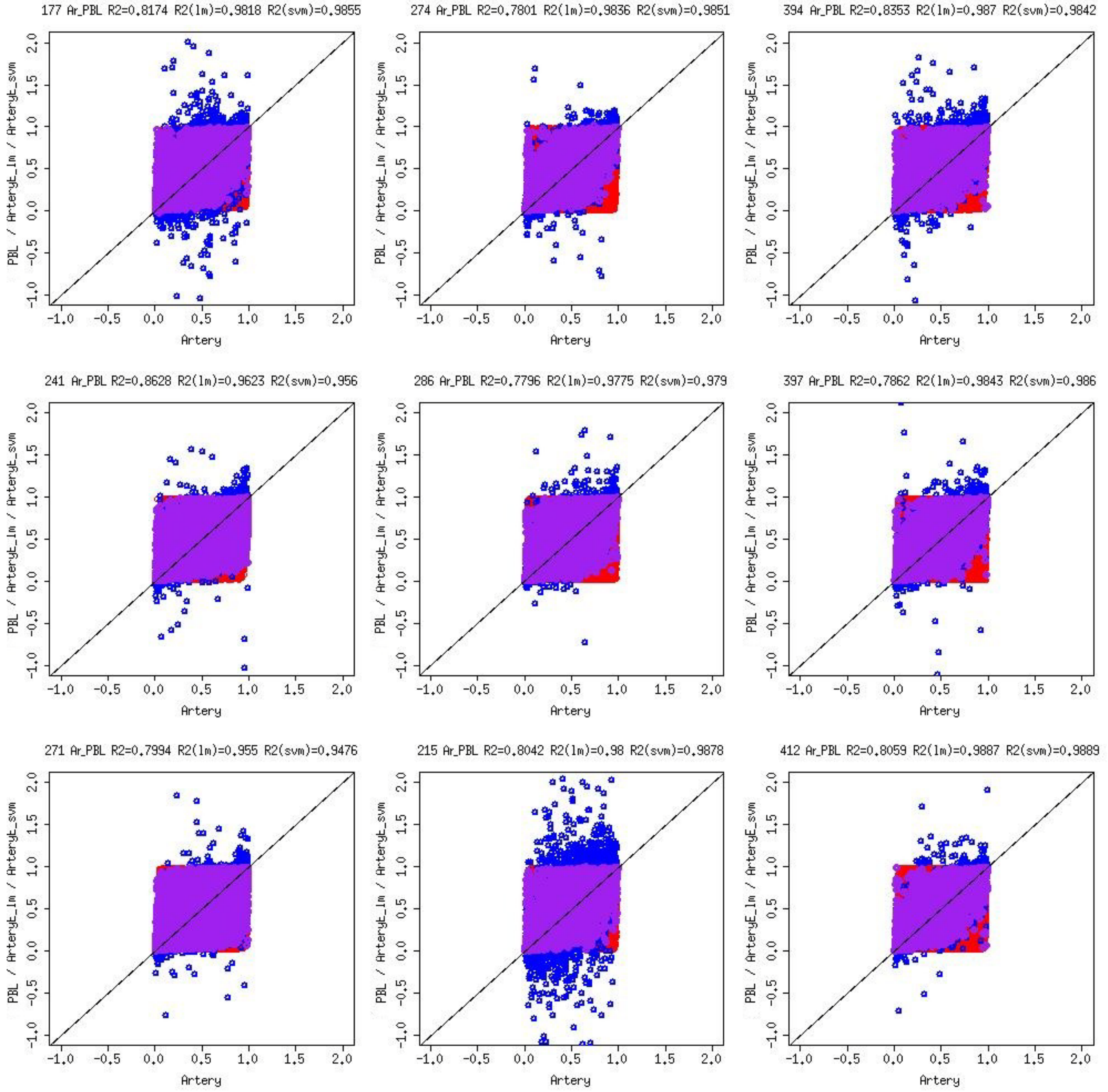

(The red circles represent PBL and Artery, the blue circles represent ArteryE_lm and Artery, the purple circles represent ArteryE_svm and Artery, ArteryE_lm is the predicted Artery by using linear regression model, ArteryE_svm is the predicted Artery by using svm model)

Supplementary S12. Scatter plot of PBL, Atrium and predicted Atrium (LM, SVM) of sample 1 to 9

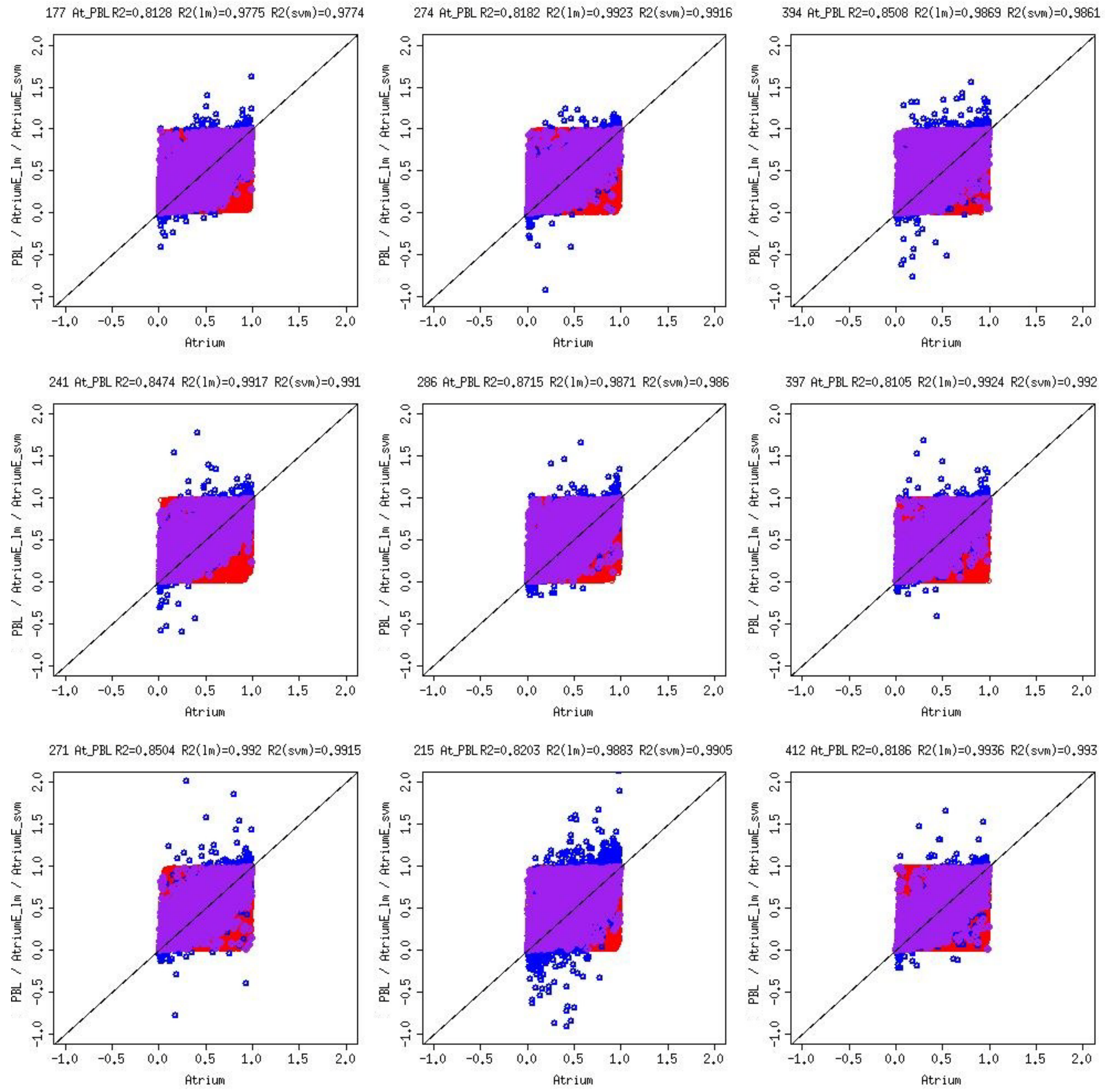

(The red circles represent PBL and Atrium, the blue circles represent AtriumE_lm and Atrium, the purple circles represent AtriumE_svm and Atrium, AtriumE_lm is the predicted Atrium by using linear regression model, AtriumE_svm is the predicted Atrium by using svm model)

Supplementary S13. Scatter plot of LCL, PBL and predicted PBL (LM, SVM) of sample 1 to 9

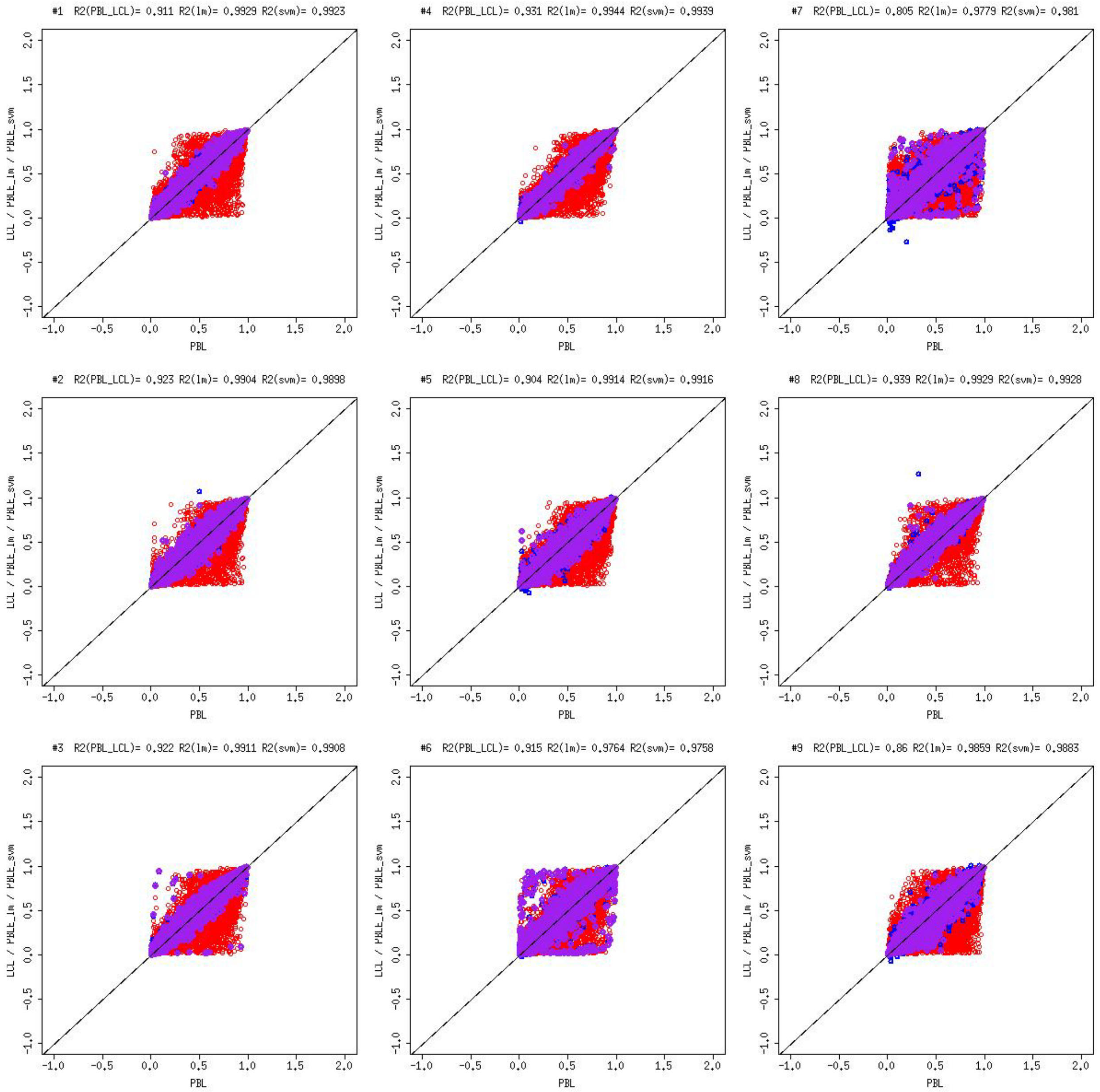

(The red circles represent LCL and PBL, the blue circles represent PBLE_lm and PBL, the purple circles represent PBLE_svm and PBL, PBLE_lm is the predicted PBL by using linear regression model, and PBLE_svm is the predicted PBL by using svm model)

Supplementary S14. Scatter plot of PBL, Artery and predicted Artery (LM, SVM) of sample 215

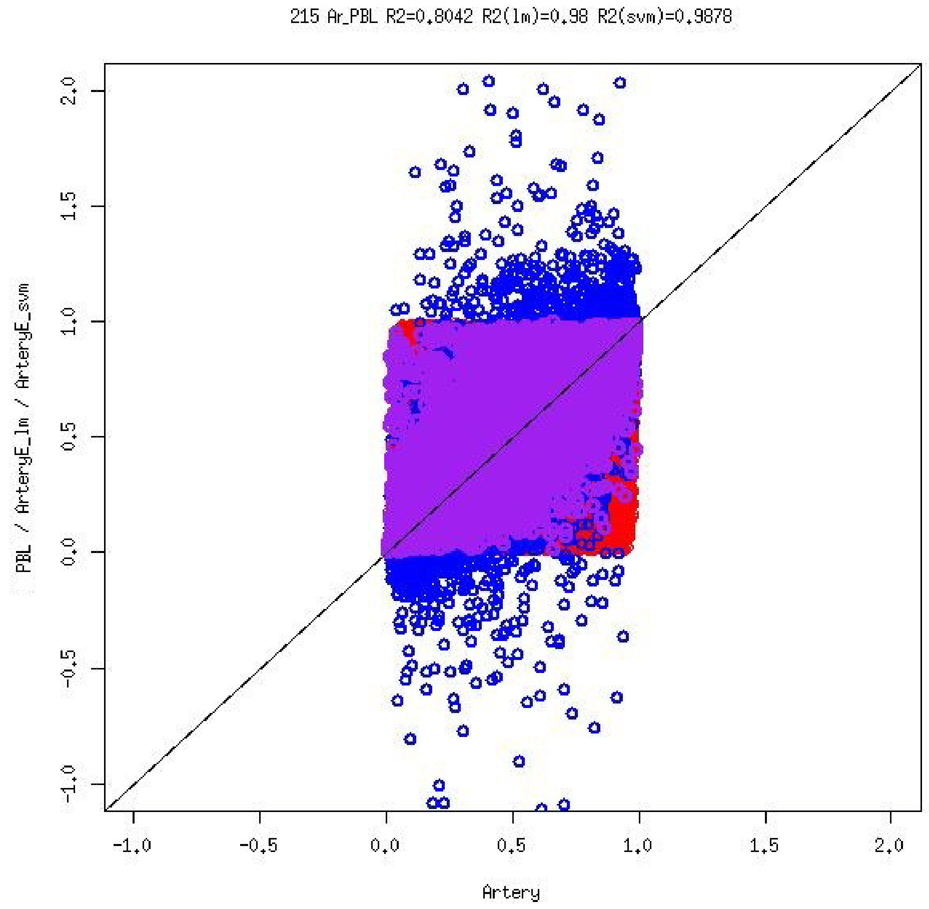

(The red circles represent PBL and Artery, the blue circles represent ArteryE_lm and Artery, the purple circles represent ArteryE_svm and Artery, ArteryE_lm is the predicted Artery by using linear regression model, ArteryE_svm is the predicted Artery by using svm model.)

Supplementary S15. Scatter plot of PBL, Atrium and predicted Atrium (LM, SVM) of sample 215

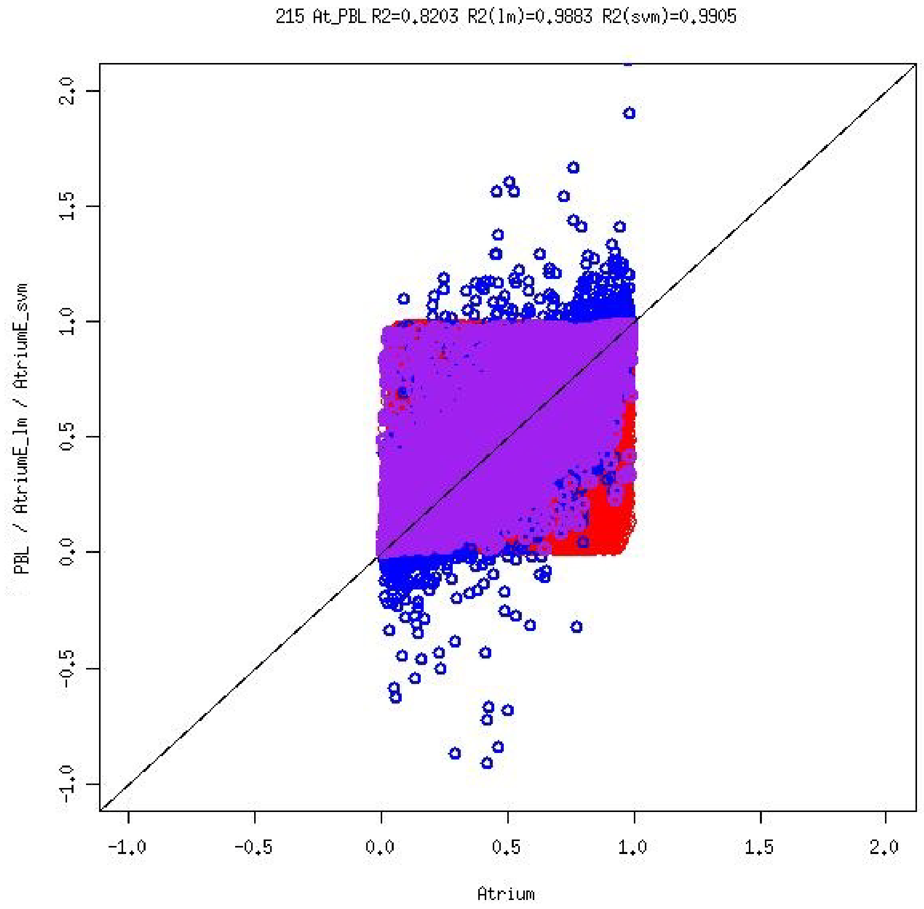

(The red circles represent PBL and Atrium, the blue circles represent AtriumE_lm and Atrium, the purple circles represent AtriumE_svm and Atrium, AtriumE_lm is the predicted Atrium by using linear regression model, AtriumE_svm is the predicted Atrium by using svm model.)

Supplementary S16. Scatter plot of LCL, PBL and predicted PBL (LM, SVM) of sample 8

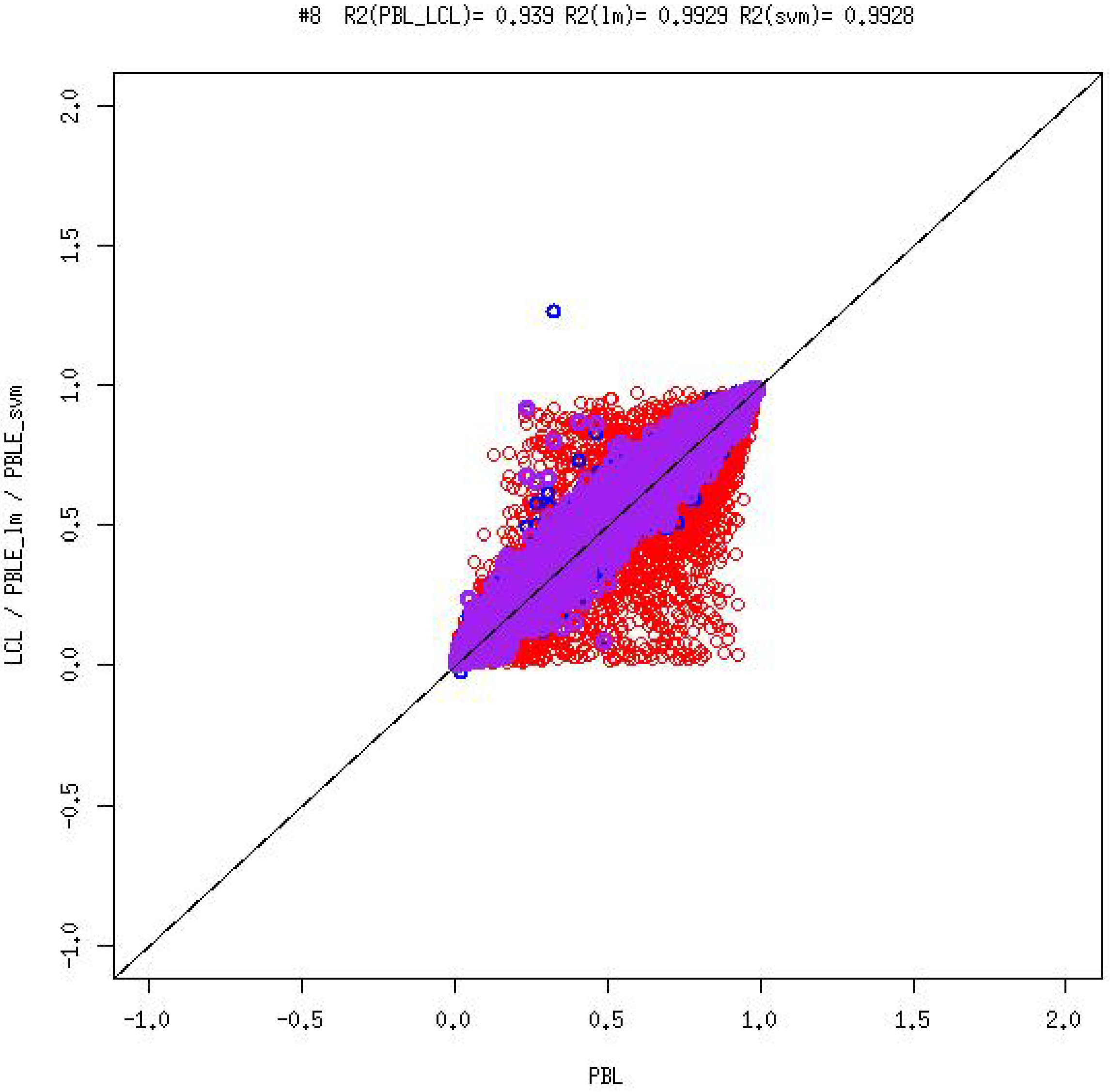

(The red circles represent LCL and PBL, the blue circles represent PBLE_lm and PBL, the purple circles represent PBLE_svm and PBL, PBLE_lm is the predicted PBL by using linear regression model, and PBLE_svm is the predicted PBL by using svm model.)

Supplementary S17. R2 of LCL-PBL dataset and GSE26211 dataset

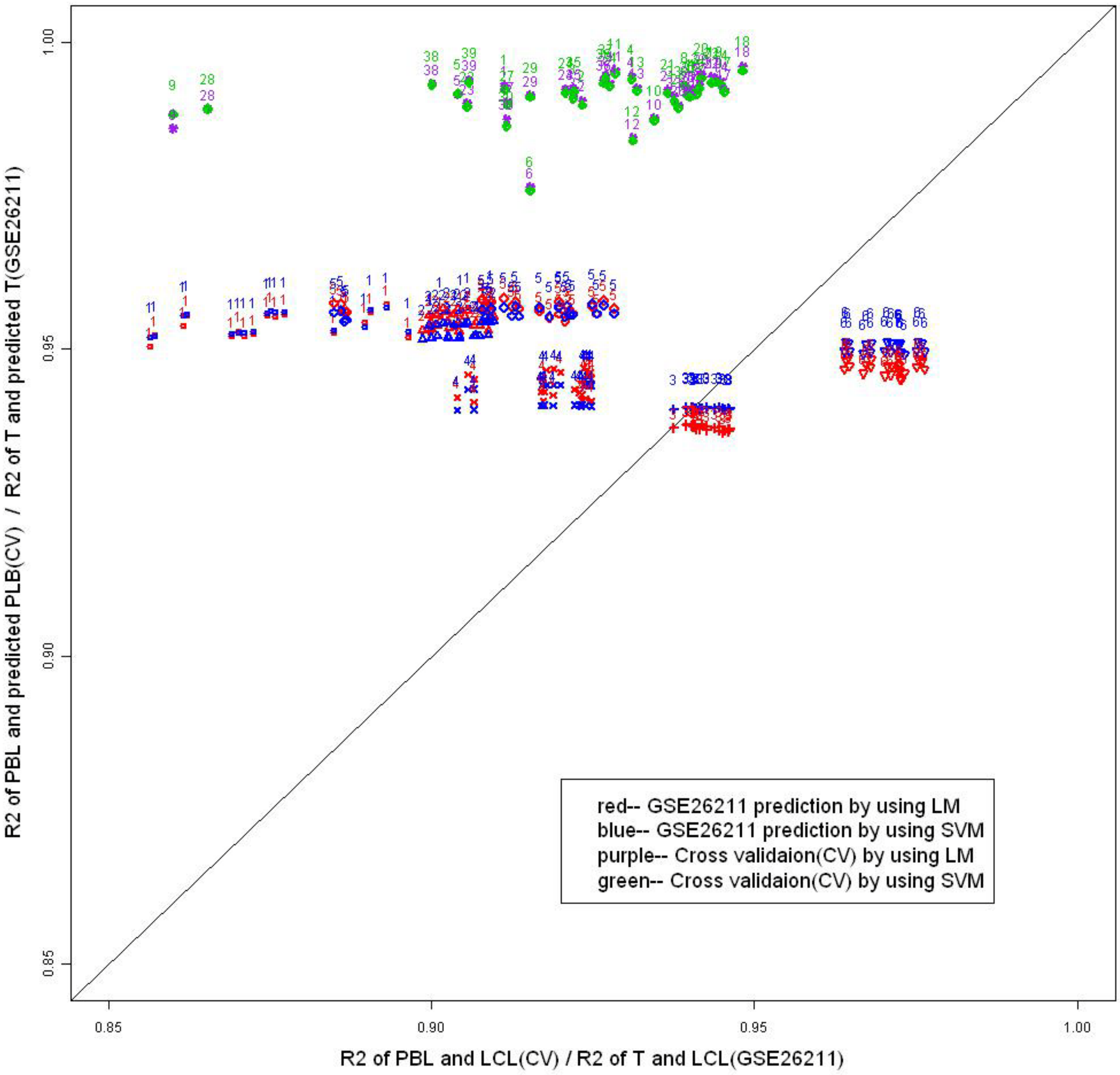

(The red and blue points indicate the 6 individuals in GSE26211, each individual is labeled by number from 1 to 6 according to GSE26211, we predict T methylation in GSE26211by using the model of the 39 samples LCL-PBL dataset. The purple and green points indicate the 39 samples of LCL and PBL by Cross Validation, each individual is labeled by number from 1 to 39.)

Supplementary S18. Sample size effect on individual specific prediction error

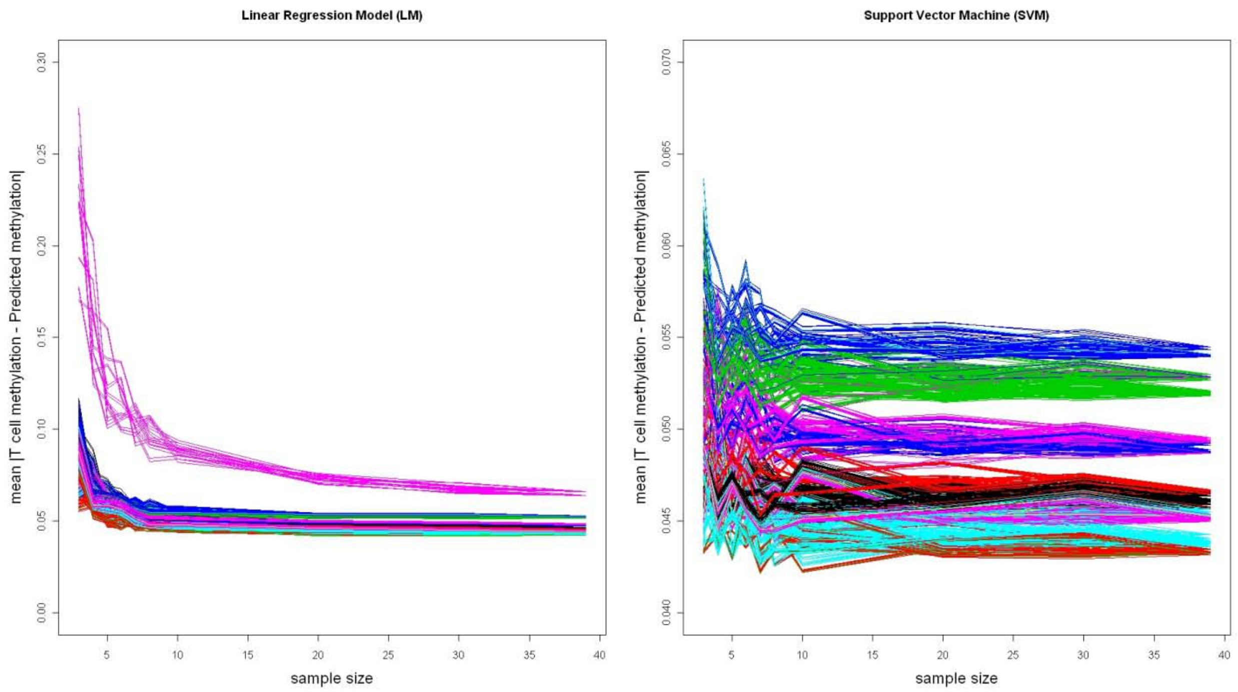

(Each line represents each LCL-T pairs in one replication, there are 10 replications for each sample size, and there are 1440 lines in total. LCL-T pairs from the same individual are in same color: individual 1- black; individual 2- red; individual 3- green; individual 4- blue; individual 5- light blue; individual 6- purple.)
